# Emerging Multimodal Strategies for Bacterial Biofilm Eradication: A Comprehensive Review

**DOI:** 10.3390/microorganisms13122796

**Published:** 2025-12-08

**Authors:** Pamela Hindieh, Joseph Yaghi, Jean Claude Assaf, Ali Chokr, Ali Atoui, Nikolaos Tzenios, Nicolas Louka, André El Khoury

**Affiliations:** 1 Centre d’Analyses et de Recherche (CAR), Unité de Recherche Technologies et Valorisation Agro-Alimentaire (UR-TVA), Laboratoire de Mycologie et Sécurité des Aliments (LMSA), Faculté des Sciences, Campus des Sciences et Technologies, Université Saint-Joseph de Beyrouth, Mar Roukos, Matn, Beirut P.O. Box 17-5208, Lebanon; 2 Ecole Doctorale “Sciences et Santé”, Campus des Sciences Médicales et Infirmières, Université Saint-Joseph de Beyrouth, Riad El Solh, Beirut P.O. Box 17-5208, Lebanon; 3 Department of Chemical Engineering, Faculty of Engineering, University of Balamand, Tripoli P.O. Box 100, Lebanon; 4 Research Laboratory of Microbiology (RLM), Department of Life and Earth Sciences, Faculty of Sciences I, Lebanese University, Hadat Campus, Beirut P.O. Box 6573, Lebanon; 5 Platform of Research and Analysis in Environmental Sciences (PRASE), Doctoral School of Sciences and Technologies, Lebanese University, Hadat Campus, Beirut P.O. Box 6573, Lebanon; 6 Faculty of Public Health, Charisma University, London EC1V 7QE, UK

**Keywords:** biofilm, extracellular polymeric substance (EPS), biofilm eradication, antimicrobial resistance, antibiofilm agents

## Abstract

Bacterial biofilms pose significant challenges in clinical, industrial, and environmental settings due to their inherent resistance to antimicrobial agents and host immune responses. Encased within a self-produced extracellular polymeric substance (EPS) matrix, these structured microbial communities demonstrate exceptional resilience, resisting conventional antimicrobial treatments and adapting to, as well as recovering from, environmental and therapeutic stresses, necessitating the development of novel anti-biofilm strategies. This review provides a comprehensive synthesis of biofilm formation, resistance mechanisms, and current and emerging approaches for controlling biofilms, with a primary focus on advancements made over the last decade. Chemical, physical, and biological strategies, including enzymatic degradation, natural compounds, chelating agents, nanoparticles, photodynamic therapy, and probiotics, have demonstrated promising antibiofilm activity. Additionally, combination therapies and targeted drug delivery systems have emerged as viable solutions to enhance the eradication of biofilms. Despite these advancements, challenges such as cytotoxicity, bacterial adaptation, and clinical applicability remain. Addressing these hurdles requires interdisciplinary research to refine existing strategies and develop innovative solutions for effective biofilm management.

## 1. Introduction

Since the discovery of microorganisms, various research, experiments, and analyses have significantly advanced science and technology. In the seventeenth century, Antonie van Leeuwenhoek first identified microbes in the calculus on his teeth [1]. These deposits contained various “animalcules” now known as dental plaque bacteria. This formation of dental coatings is one of the earliest documented bacterial biofilms [2]. Biofilm formation is a strategy used by microorganisms to enhance their survival in hosts and harsh environments [3]. Although bacteria have a general tendency to live in a biofilm.

When faced with unfavorable conditions (such as desiccation, shear stress, toxic compounds, and protozoan grazing), bacteria can shift from a free-floating (planktonic) state to a sessile state, allowing them to adhere, grow, and form communities on surfaces [4].

**Figure 1:**
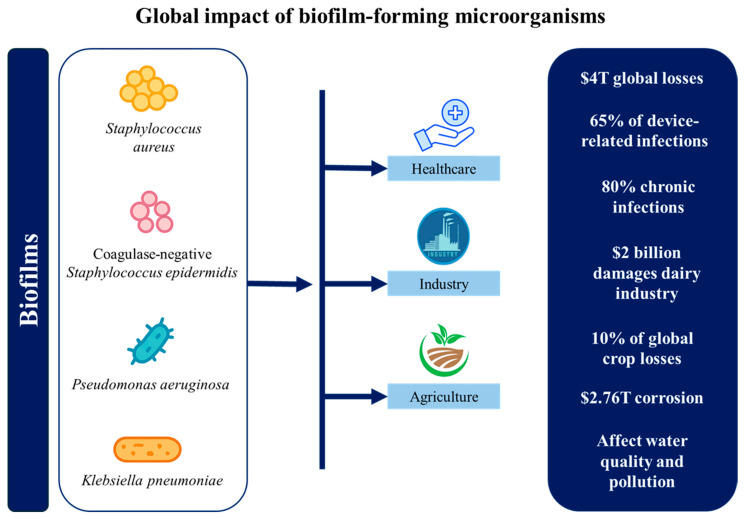
Summary of the global impact of biofilm-forming microorganisms.

Biofilms are an organized, three-dimensional community of microorganisms that adhere to biotic and abiotic surfaces and are encased in a self-produced extracellular substance (EPS) matrix [5]. These intricate structures, first described in detail by Ref. [6] in 1978, have since been recognized as a predominant form of microbial life in various environments. Within the biofilm matrix, organisms are arranged rather than scattered randomly and regulated by several genes [7]. Both homogeneous and heterogeneous biofilms are possible. A homogeneous biofilm consists of a single microbial species, whereas a heterogeneous biofilm includes different species living together. Common biofilm-forming bacteria include *Pseudomonas aeruginosa*, *Staphylococcus epidermidis*, *Escherichia coli*, and *Staphylococcus aureus*, with mixed-culture biofilms often demonstrating enhanced stability through interspecies interactions [8]. Indeed, these dynamic and complex structures offer remarkable protection to enclosed microbial communities against a wide array of environmental challenges. These include resistance to various biocides and antibiotics used in industrial and clinical settings, UV damage, metal toxicity, anaerobic conditions, acid exposure, salinity fluctuations, desiccation, and bacteriophages [9]. Furthermore, biofilms shield bacteria from mechanical stress, shear forces, and the host’s immune cells, while enabling them to endure external stressors like nutrient scarcity and osmolarity changes [10]. Thus, biofilms are ubiquitous due to this multifaceted protection mechanism, coupled with the biofilm’s ability to control various metabolic processes. They represent a fundamental microbial survival strategy in diverse environments, from soil and aquatic ecosystems to industrial piping systems, indwelling medical devices, and live tissues such as tooth enamel, heart valves, lungs, and middle ears. However, their impact extends beyond these everyday occurrences [11]. Biofilms can be neutral, harmful, or good. While biofilms that form on open wounds after infection are dangerous, biofilms that are a part of the natural ecosystem are neutral. Biofilms may help address oil spill-related ground contamination [12]. Consequently, the significance of biofilms extends across various fields, encompassing healthcare, industrial processes, and ecological studies, thereby making them a critical focus of interdisciplinary scientific research. Figure 1 illustrates a concise overview of the principal ways in which biofilm-forming microorganisms negatively influence human health, industrial processes, and environmental systems in daily life.

In medical settings, both device-related and non-device-related biofilm infections are common worldwide and cause many fatalities every year [13]. Biofilms cause over $4 trillion in global economic losses annually, impacting healthcare, infrastructure, agriculture, and energy sectors [14]. These biofilms pose significant challenges in treating chronic infections, particularly those associated with implanted medical devices such as heart valves, catheters, joint prostheses, intrauterine devices, orthopedic implants, cardiac pacemakers, and contact lenses [12,15,16]. The clinical impact of biofilm formation on medical devices is substantial, with approximately 65% of device-related infections attributed to biofilms. Therefore, these infections can lead to severe complications, often necessitating device removal and prolonged antimicrobial therapy [17]. For example, catheter-associated urinary tract infections (CAUTIs) are frequently caused by biofilm-forming pathogens like *E. coli*, *S. aureus*, and *P. aeruginosa*, leading to complications in hospitalized patients [18]. Other common biofilm-associated pathogens include *Enterobacteriaceae*, coagulase-negative *staphylococci*, *Acinetobacter* spp., and *Enterococcus* spp. [19]. Additionally, scanning electron microscopy has revealed that most indwelling central venous catheters are colonized by these aggregates of microorganisms embedded in a biofilm matrix. Among these, staphylococci are the leading cause of biofilm-associated infections, with highly virulent *S. aureus* strains frequently causing severe localized infections or sepsis [20]. These infections can result in bloodstream infections and device failure in hospitalized patients [21]. Beyond catheters, biofilms are also implicated in prosthetic heart valve infections [22,23] and contribute to periodontal diseases and tooth decay through dental plaque formation [24]. Additionally, *P. aeruginosa* and *S. aureus* biofilms have been extensively reported in the context of persistent wound infections and respiratory tract infections in cystic fibrosis patients [25,26]. In healthcare, biofilm-related chronic wounds, lung infections, prosthetic joint failures, catheter infections, and antimicrobial resistance contribute billions in costs annually [27]. According to the National Institutes of Health (NIH), biofilms are responsible for approximately 80% of chronic infections and numerous pathogen outbreaks in healthcare settings [21]. The challenge is further intensified by the emergence and global spread of multidrug-resistant (MDR) bacteria, defined as organisms resistant to at least one agent in three or more antimicrobial categories [28]. These MDR pathogens, including *P. aeruginosa*, *S. aureus*, *K. pneumoniae*, and *A. baumannii*, are frequently implicated in biofilm-related device infections and chronic wounds, and their resistance severely limits therapeutic options [29]. Their resistance to antimicrobial agents, disinfectants, and immune responses makes them particularly difficult to eradicate, with biofilm-embedded bacteria being up to 1000 times more resistant to antibiotics than planktonic cells [30]. This resistance exacerbates the global antimicrobial resistance crisis, which has contributed to an estimated 4.71 million deaths worldwide, with biofilms representing a significant and persistent factor in this burden [31]. Industrial sectors face substantial economic losses due to biofilm-related issues. Microbial corrosion alone accounts for around $2.76 trillion annually, significantly impacting infrastructure [32]. Different studies have described how biofilms cause biofouling in industrial equipment [33], leading to decreased efficiency, increased energy consumption, and accelerated material degradation [34]. A prime example is the formation of biofilms in water distribution systems, where they can harbor pathogens like *P. aeruginosa*, posing public health risks [35]. Biofilm contamination further escalates food safety expenses and disrupts water and energy systems, creating widespread economic and environmental challenges. Although in the food industry, biofilms can have both positive and negative effects. On the positive side, biofilms can be used in wastewater treatment to degrade pollutants, in biofuel production, and for the filtration of drinking water [36]. Nonetheless, biofilms can play a crucial role in fermentation processes, contributing to improved flavor and texture in products such as yogurt and cheese [37]. These biofilms often involve beneficial microorganisms, such as lactic acid bacteria (LAB) and yeast, which are essential for traditional fermented foods like cheese, vinegar, kombucha, kefir, wine, lambic beer, miso, and kimchi [38]. However, biofilms in the food industry can contaminate food products, leading to foodborne illnesses. Those formed by major foodborne pathogens such as *E. coli*, *Listeria monocytogenes*, *Salmonella* spp., and *Campylobacter jejuni* pose a particular risk, contributing to persistent contamination and food safety concerns [39]. In agriculture, biofilms contribute 10% of global crop losses and cause $2 billion in dairy industry damages annually [27]. Furthermore, in urban contexts, biofilms play dual roles. They can actively participate in wastewater treatments, organic matter decomposition, nutrient dynamics, and biogeochemical cycling, being a key component of ecosystem functioning [40,41]. However, they can also be detrimental, where biofilms in water distribution systems can harbor pathogenic organisms and contribute to the deterioration of water quality [42]. Additionally, biofilm formation on microplastics can enhance the sorption of hydrophobic organic compounds (HOCs), potentially increasing their transfer through food chains and contributing to ecological pollution [43]. Biofilms also serve as reservoirs for antibiotic resistance genes (ARGs), facilitating the spread of antimicrobial resistance among microbial communities in aquatic ecosystems exposed to anthropogenic pollutants like wastewater treatment plant effluents [44].

While numerous reviews have summarized biofilm biology and conventional control measures, this article uniquely focuses on emerging multimodal strategies for biofilm eradication developed over the last decade. We highlight innovative chemical, physical, and biological interventions, such as enzymatic degradation, natural compounds, nanoparticles, aPDT, CAP, and phage–antibiotic combinations, emphasizing their mechanisms, advantages, and limitations. Furthermore, this review integrates recent findings on combination therapies and targeted delivery systems, providing insights into overcoming persistent biofilm resistance. By synthesizing these advances and identifying ongoing challenges, this review seeks future research directions in strategic biofilm control.

## 2. Methodology and Use of AI-Assisted Tools

This narrative review was based on peer-reviewed literature retrieved from major scientific databases and online research platforms. Articles were selected according to their relevance to the topic and scientific quality without date restrictions. The GenAI tool Napkin AI was used exclusively to generate illustrative charts for the visual representation of concepts. No text, interpretation, or scientific content was created by the tool. All figures were manually checked, edited, and finalized by the authors in accordance with MDPI’s GenAI transparency policy.

## 3. Biofilm Structure

Biofilms are highly organized microbial communities composed primarily of water (up to 90%) and microbial mass [45]. Their structure consists of microcolonies of bacterial cells embedded within an EPS matrix, interconnected by water channels that facilitate the diffusion of nutrients, enzymes, metabolites, oxygen, and waste products [46,47]. The basal layer of the biofilm consists of a hydrated mixture of polysaccharides, proteins, extracellular DNA (eDNA), lipids, and enzymes, which contribute to biofilm stability and functionality [48,49]. The EPS matrix, also known as the glycocalyx, constitutes 75–95% of the biofilm’s dry mass, with polysaccharides such as poly-N-acetylglucosamine (PNAG), alginate, amylose-like glucan, cellulose, and galactosaminogalactan comprising 50–90% of its organic components [8,50]. These polysaccharides form a dense, mesh-like matrix stabilized by intermolecular interactions among their hydroxyl groups [51]. The EPS matrix varies in thickness from nanometers to hundreds of micrometers, acting as a structural scaffold that supports cell adhesion, cohesion, and protection against environmental stressors [52]. Its ionic composition plays a crucial role in maintaining biofilm integrity, with positively charged ions such as Ca^2+^ and Mg^2+^ cross-linking polymers, increasing resistance to shear forces, and allowing biofilms to reach thicknesses of up to 300 μm [53]. The chemical nature of EPS differs between bacterial groups: in Gram-negative bacteria, it is often neutral or polyanionic, incorporating uronic acids like D-glucuronic acid to bind divalent cations [54], whereas Gram-positive bacteria, such as *Staphylococci*, are predominantly cationic, rich in teichoic acid with minimal protein content [55]. This diverse composition and structural organization are essential for maintaining biofilm architecture and functionality. As a result, biofilms form a highly viscoelastic, rubber-like structure, enhancing their resilience and stability. Moreover, sessile bacteria within a biofilm differ from planktonic cells, exhibiting distinct growth patterns, gene expression, transcription, and translation rates [56]. Their ability to adapt to microenvironments with high osmolarity, limited nutrients, and increased cell density allows them to develop specialized functional traits [57].

### 3.1. Biofilm Formation Process and Lifecycle

As mentioned before, biofilms can be described as intricate communities of microorganisms embedded within a self-produced EPS. The dense association of microorganisms within a biofilm creates a unique microenvironment that fosters the development of nutrient availability gradients, genetic exchange, and quorum sensing (QS) mechanisms. Although the biofilms generated by various bacteria have many characteristics, they can also differ slightly depending on the species.

**Figure 2:**
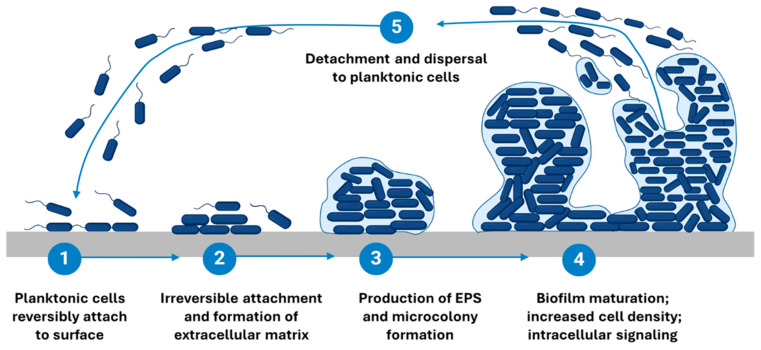
Stages of biofilm formation. Schematic representation of the multi-step biofilm formation process. (1) Reversible attachment of planktonic microorganisms to the surface. (2) Irreversible adhesion and bacterial aggregation. (3) Microcolony formation and EPS production, leading to matrix development. (4) Biofilm maturation, resulting in a structured three-dimensional community. (5) Detachment and dispersal, allowing bacteria to revert to a planktonic state and establish biofilms in new locations. Created on BioRender.com, https://app.biorender.com/illustrations/67b34f1adf448d5d0716f8d5?slideId=936aac43-f7be-4db8-aca2-e0a65cb6c8af*,* accessed on 18 February 2025.

The formation of the three-dimensional architecture of biofilm is a several-step process involving multiple stages and factors that transform free-floating (planktonic) bacteria into a highly organized, surface-attached community. It consists of five main phases presented in Figure 2: reversible (1) and irreversible (2) surface adhesion, proliferation, microcolony formation (3), maturation (4), and the dispersion/detachment (5) of planktonic cells or EPS-included cell aggregates.

The initial reversible attachment phase typically occurs within the first hours, followed by irreversible adhesion over 2–6 h, during which microorganisms adhere to a surface and begin to establish microcolonies [58]. Proliferation and microcolony formation usually develop within 6–24 h, while early maturation is commonly observed between 24–48 h [59]. A fully mature biofilm is generally reached after 48–72 h, although some species (*P. aeruginosa*, *S. aureus*) exhibit mature architectures beyond 72–96 h, depending on nutrient availability and hydrodynamic conditions [60,61]. It is important to note that these timeframes can vary significantly based on factors such as the species of microorganisms involved, the growth conditions, and the specific in vitro model employed [62]. Furthermore, the definitions of each stage, particularly that of a “mature biofilm,” are not universally standardized and can differ across studies, complicating the interpretation of biofilm research. In the following sections, we will delve into each stage of the biofilm lifecycle in greater detail, exploring the underlying mechanisms and factors that influence biofilm development.

#### 3.1.1. Initial Attachment (Reversible Surface Adhesion)

Biofilm formation begins with the reversible surface adhesion of planktonic bacteria (phase 1), where the attachment is transient and easily reversible [63]. However, a bacterium’s ability to adhere to a surface depends on various factors, including the physicochemical properties (such as roughness, hydrophobicity, and chemical composition) of the substrate, the characteristics of the bacterial cell surface, and environmental conditions like nutrient levels, pH, ionic strength, and temperature [64]. Planktonic cells initially attach to biotic or abiotic surfaces, where their stability is governed by a balance of attractive and repelling forces. These forces include hydrophobic interactions, steric forces, electrostatic forces, Van der Waals interactions, and protein adhesion [65]. The attachment process is primarily driven by Brownian motion and gravitational forces, while hydrodynamic forces in the surrounding environment further influence bacterial movement and adhesion [66]. Additionally, the velocity and direction of attachment are regulated by the bacterial cell surface composition. Motile bacteria have a distinct advantage over non-motile species, as they can actively counteract hydrodynamic and repulsive forces using flagella [51]. This has been observed in *P. aeruginosa*, *Vibrio cholerae*, *L. monocytogenes*, and *E. coli*, where flagella-driven motility enhances surface colonization [67]. If conditions are unfavorable, the initial attachment remains reversible, allowing cells to detach and revert to a planktonic state. As bacteria encounter surfaces, the intracellular signaling molecule bis-(3ʹ-5ʹ)-cyclic dimeric guanosine monophosphate (c-di-GMP) regulates their transition from free-swimming to a biofilm-forming state [68]. Initially, low c-di-GMP levels allow planktonic bacteria to move freely, but as attachment occurs, increasing c-di-GMP restricts motility and enhances EPS production, leading to irreversible adhesion and biofilm initiation [68]. However, structural modifications in bacterial surface proteins, along with increased acid–base and hydrophobic interactions, enhance bacterial adhesion by displacing interfacial water and strengthening surface contact [69]. These interactions facilitate the transition from reversible to irreversible attachment, where bacteria form a monolayer that becomes permanently adhered to the surface [70]. In some cases, bacteria can actively explore surfaces through swarming mechanisms mediated by appendages such as Type IV pili, flagella, fimbriae, or fibrillae, further contributing to biofilm establishment [71].

#### 3.1.2. Bacterial Adhesion and Aggregation (Irreversible Surface Adhesion)

The second stage of adhesion, known as the anchoring or attachment phase, involves a molecularly coordinated interaction between bacterial adhesins and the outermost layer of a substrate. During this irreversible attachment phase, bacteria undergo physiological transformations as they transition from planktonic to sessile states [72]. They may lose surface appendages and activate new metabolic pathways to support biofilm formation [73]. For example, *S. epidermidis* produces polysaccharide intercellular adhesin (PIA), which plays a crucial role in cell-to-cell adhesion and the development of stable biofilm structures [12]. Additionally, genes responsible for synthesizing and secreting EPS are upregulated, leading to the formation of small bacterial clusters on the surface [74]. As described by ref. [49], EPS forms a protective matrix around the growing bacterial community, providing structural integrity and enhancing resistance to environmental stressors. This reinforcement stabilizes bacterial adhesion, making detachment difficult without external intervention. Notably, biofilm formation is not limited to surface attachment; free-floating microbes can adhere to one another and to surface-bound cells, forming complex microbial aggregates [75]. Moreover, certain species facilitate the attachment of others, highlighting the cooperative nature of biofilm development [51].

#### 3.1.3. Proliferation and Microcolony Formation

The third phase of biofilm formation begins as bacteria proliferate and aggregate into microcolonies, driven by chemical signaling within the EPS matrix [63]. This matrix serves as a scaffold that binds cells together, enabling the three-dimensional development of the biofilm. The formation of microcolonies is facilitated by flagella- and Type IV pili-mediated motilities, promoting bacterial–surface interactions and cell–cell aggregation [76]. Notably, c-di-GMP levels can vary spatially within mature biofilms, with higher concentrations often found at outer boundaries where active growth occurs [77]. As bacterial populations grow, they form diverse micro-communities that cooperate through substrate exchange, metabolic product flow, and waste elimination, fostering syntrophic associations where metabolically distinct bacteria rely on each other to utilize specific substrates for energy [78]. Additionally, increased c-di-GMP levels can be triggered by environmental stressors such as hypochlorite exposure [79], highlighting its role in adaptive responses during biofilm development.

#### 3.1.4. Maturation

As biofilms mature (phase 4), they develop complex three-dimensional structures, often resembling mushrooms or towers, that adapt to environmental conditions [65]. This architectural complexity is facilitated by EPS, eDNA, and QS, which collectively regulate bacterial cooperation, gene expression, and metabolic adaptation [45]. The mature biofilm exhibits heterogeneity, with distinct regions displaying different metabolic activities and stress responses, contributing to its resilience [80]. Water channels within the biofilm function as a primitive circulatory system, allowing nutrient distribution and waste removal [81]. Programmed cell death plays a structural role, as dead cells serve as scaffolds, while viable cells import essential resources to sustain growth [82]. Signaling molecules secreted by bacterial cells trigger the expression of biofilm-specific genes, enhancing virulence and strengthening the biofilm matrix [83]. For example, *P. aeruginosa* secretes polysaccharides such as alginate, Pellicle (Pel), and Polysaccharide Synthesis Locus (Psl), which reinforce biofilm stability, while eDNA stabilizes cell-to-cell interactions in early biofilm formation [84]. Additionally, surfactants and modulins, as observed in *Staphylococcus* species, contribute to biofilm maturation through QS-mediated mechanisms [85]. Structurally, mature biofilms consist of three distinct layers: an inner regulatory layer, a microbial basement layer, and an outer layer where planktonic cells prepare for dispersal [86]. Within mature biofilms, a subpopulation of cells may enter the Viable But Non-Culturable (VBNC) state, characterized by metabolic dormancy and resistance to environmental stress [29]. The maturation process significantly increases biofilm resistance, making bacterial communities more tolerant to antibiotics and other environmental stressors than their planktonic counterparts.

#### 3.1.5. Dispersion/Detachment

Dispersion (phase 5), the final stage of biofilm development, involves the detachment of microbial cells, either as individual planktonic bacteria or as aggregates encased in EPS [87]. This process can be triggered by environmental factors such as nutrient availability, oxygen fluctuations, and the accumulation of signaling molecules [88]. Bacterial enzymes contribute to dispersion by breaking down the EPS matrix, freeing the cells to migrate and colonize new surfaces, thus restarting the biofilm lifecycle [89]. Detachment can occur actively through enzymatic degradation, QS, and gene regulation, or passively due to external forces like fluid shear and abrasion [90]. As biofilms mature, nutrient depletion and metabolic waste accumulation further drive dispersal, enabling bacteria to escape unfavorable conditions and establish new biofilms [91]. Recent studies have identified specialized “scout cells” within mature biofilms that exit the biofilm early during dispersal [90]. These metabolically active cells explore new niches and, upon finding favorable conditions, can signal remaining cells to follow, initiating colonization [92]. This behavior reflects a strategic and coordinated dispersal mechanism that enhances survival and adaptability. The primary dispersion mechanisms include erosion, the gradual release of single cells or small clusters; sloughing, the sudden detachment of large biofilm sections; and seeding, the rapid release of bacterial clusters from the biofilm interior [93]. Dispersion has become a key focus in biofilm control strategies due to its significance in bacterial persistence and pathogenicity.

Understanding the biofilm formation, lifecycle, and structure provides insights into the development of effective anti-biofilm strategies. For instance, targeting the mechanisms of initial attachment could prevent biofilm formation altogether, while disrupting the EPS matrix could compromise the integrity of established biofilms. Similarly, inducing dispersion could render biofilm cells more susceptible to conventional treatments. As we delve deeper into current strategies for biofilm dispersion in subsequent sections, this foundational knowledge will provide crucial context for understanding the mechanisms and efficacy of various antibiofilm approaches.

## 4. Mechanisms of Biofilm Resistance

The exceptional biofilm’s resistance to antimicrobial agents and host immunological response is a key explanation of their persistence and the challenges they pose in various daily contexts. This resistance is complicated, resulting from several interconnected mechanisms that collectively contribute to the survival of biofilm-associated bacteria under adverse conditions. The challenge is further compounded when the causative organisms are MDR strains, which possess additional resistance mechanisms. The co-existence of biofilm-mediated and genetically encoded multidrug resistance creates barriers to successful infection management.

**Figure 3:**
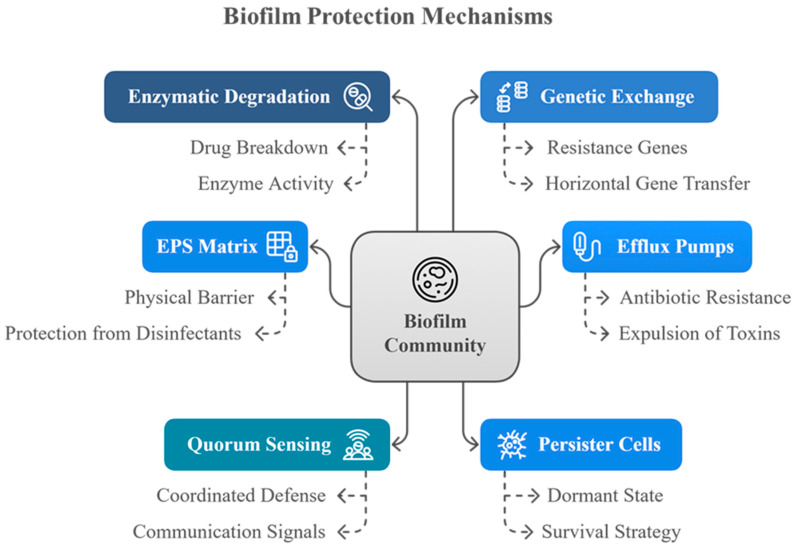
Mechanisms of biofilm protection.

Figure 3 illustrates the diverse defense mechanisms that biofilm communities employ to survive antimicrobial challenges.

This diagram illustrates the multifaceted defense strategies employed by biofilm communities. Key protective mechanisms include enzymatic degradation of antimicrobial agents, genetic exchange facilitating resistance gene transfer, efflux pumps that expel harmful substances, the EPS matrix acting as a physical barrier, quorum sensing for coordinated defense, and the presence of persister cells that enter a dormant state to withstand hostile conditions. These mechanisms collectively enhance the biofilm’s resistance to antibiotics and disinfectants.

### 4.1. Physical Barrier

The extracellular polymeric substance (EPS) matrix is a major determinant of biofilm resistance, acting as a multifaceted physical and chemical barrier that limits immune activity and antimicrobial penetration [94]. It enhances bacterial survival by restricting the penetration and diffusion of antimicrobial compounds, reducing their concentration within the biofilm, and giving bacterial cells more time to develop tolerance [95]. Its permeability varies with biofilm composition, age, and structural density rather than a single molecular-weight cutoff [96]. Small solutes, including many antibiotics, generally diffuse at 75–90% of free-water rates, whereas larger macromolecules and particles face increasing restriction [97]: structures ≲40–70 nm penetrate more readily, while those ≥100 nm often require water channels or local matrix rearrangements [98]. This reflects the molecular diversity of the EPS, composed of proteins (~10 kDa to >600 kDa), polysaccharides (<1–6 kDa), and humic-like substances (1–6 kDa), which together create a heterogeneous molecular sieve that imposes steric and physicochemical barriers to diffusion [99,100]. In addition to size-dependent exclusion, the EPS also binds to charged antimicrobial peptides, limits oxidative stress from phagocytes, blocks surface immunoglobulins, and inhibits complement binding [101]. Additionally, it secures the biofilm to surfaces, traps nutrients, provides structural support, facilitates quorum sensing for cell-to-cell communication, and enables horizontal gene transfer, promoting genetic adaptability [102]. Studies have shown that EPS-deficient mutants are more susceptible to antimicrobial agents, while EPS inhibition reduces cell adhesion and drug tolerance [103]. Furthermore, biofilm-associated enzymes contribute to passive resistance by neutralizing antimicrobial molecules. For instance, catalase enzymes in *S. epidermidis* biofilms enhance resistance to physicochemical agents, and eDNA can bind to aminoglycoside antibiotics, decreasing their effectiveness [104]. Collectively, these factors illustrate how the EPS matrix functions as both a physical barrier and a biochemical shield, contributing significantly to the persistence and treatment difficulty of biofilm-associated infections.

### 4.2. Metabolic Dormancy/Persister

A critical aspect of biofilm resistance is the metabolic dormancy exhibited by a subset of cells within the biofilm, particularly in deeper layers, where restricted oxygen and nutrient flow lead to reduced metabolic activity and slowed growth [105]. Biofilm-associated bacteria exhibit high levels of antibiotic tolerance due to factors such as reduced growth rate, stress responses, and the presence of persister cells [106]. These cells are a small, dormant subpopulation that evade antibiotic action by downregulating cellular metabolism through toxin–antitoxin systems [107]. Because most antibiotics target actively growing cells, persisters remain largely unaffected, surviving high concentrations of antimicrobial agents and later reviving to repopulate the biofilm, leading to recurring infections [108]. Unlike genetically resistant mutants, persisters are phenotypic variants that do not acquire antibiotic resistance genes but create conditions that facilitate the emergence of resistant strains [109]. Their tolerance to antibiotics is influenced by reversible changes induced by starvation, environmental stress, and adaptive responses such as the SOS and stringent responses [110]. In addition to persisters, VBNC cells represent another dormant phenotype contributing to biofilm persistence. VBNC cells enter a low-metabolic, non-dividing state in response to environmental stresses such as nutrient limitation, oxidative damage, or antimicrobial pressure, yet retain viability and the potential for resuscitation [29]. These cells can evade detection in culture-based diagnostics and reawaken under favorable conditions, posing significant risks for relapse and chronic infection. Furthermore, scout cells are a specialized subpopulation that sense environmental cues, reactivate metabolism, and initiate early detachment from the biofilm, potentially reseeding new biofilms and promoting infection spread [90]. Collectively, persister cells, VBNC cells, and scout cells represent key phenotypic variants within biofilms that ensure survival under hostile conditions and complicate eradication efforts. As a result, research efforts are increasingly focused on understanding these subpopulations to develop antibacterial strategies that specifically target and eliminate these resilient cells.

### 4.3. Efflux Pumps

Efflux pumps are proteinaceous transporter machinery systems localized within the microorganism’s cell membrane. These pumps actively force out a wide range of antibiotics and toxic compounds from bacterial cells, reducing intracellular drug concentrations and promoting cell survival [111]. Furthermore, the expression of efflux pump genes is often upregulated in biofilms, leading to rapid export of antimicrobial compounds and decreased therapeutic efficacy [112]. These transporters demonstrate remarkable diversity, capable of handling either multiple structurally diverse substances (including drugs, toxins, and metabolites) or specialized single substrates [113]. They work in conjunction with other resistance mechanisms, such as reduced biofilm permeability and persister cell formation, to establish a robust defense system. Beyond their role in antimicrobial resistance, efflux pumps support quorum sensing, which regulates biofilm formation and maintenance [114]. They also contribute to cellular homeostasis by exporting harmful byproducts and facilitating adaptation through genetic upregulation, strengthening overall resistance mechanisms [115]. Targeting these pumps through inhibitors or regulatory pathway disruption presents a promising therapeutic strategy for combating biofilm-associated infections, potentially improving treatment outcomes by compromising their defense systems.

### 4.4. Quorum Sensing

Quorum sensing (QS) is a critical mechanism by which bacteria within biofilms communicate through the release and detection of small signaling molecules known as autoinducers (AI) [116]. The chemical structures of the autoinducers differ among microbial species. They can be generally classified as acyl-homoserine lactones (AHLs), autoinducing peptides (AIPs), or the broadly recognized autoinducers-2 and 3 (AI-2 and AI-3), which function across Gram-negative and Gram-positive bacteria [117]. Continuously produced and released by bacterial cells, AIs accumulate extracellularly as cell density increases [118]. When their concentration reaches a critical threshold, the quorum level, autoinducer receptor binding triggers synchronized gene expression changes [118]. This synchronized behavior enables bacteria to function as a cohesive unit, influencing key aspects of biofilm dynamics, including cellular proliferation, macro-colony formation, EPS production, structural integrity, biofilm dispersal, and overall development [119]. QS also regulates genes involved in virulence expression, metabolic activity coordination, and stress responses [120]. Ultimately, QS contributes significantly to the increased resistance of mature biofilms against mechanical stress, harsh environmental conditions, and antimicrobial agents [121].

### 4.5. Heterogeneity and Genetic Diversity

Biofilms exhibit metabolic and physiological heterogeneity due to nutrient and oxygen gradients within their structure [122]. Surface bacteria are metabolically active and more vulnerable to antibiotics, while those in the deeper layers face oxygen depletion and nutrient scarcity, leading to reduced metabolism and enhanced drug tolerance [123]. This stratification promotes the emergence of persister cells, small colony variants (SCVs) characterized by their slow growth and altered metabolism. These variants emerge within biofilms and contribute to their persistence, making infections more difficult to eradicate. The polymicrobial nature of biofilms further contributes to survival, as the interaction between species allows non-resistant bacteria to tolerate antibiotics through protective biofilm components and horizontal gene transfer (HGT) [124]. The proximity of cells, coupled with reduced shear forces, facilitates frequent plasmid exchange, accelerating the spread of ARGs [125]. Biofilms also act as reservoirs for ARGs, which can spread through conjugation, transformation, and transduction, ensuring the stability and adaptability of microbial communities [126]. The genetic and phenotypic diversity within biofilms allows rapid adaptation to environmental stressors, with subpopulations in varying physiological states [127]. For example, localized pH changes or enzymatic activity can deactivate antibiotics, while SCVs in *P. aeruginosa* biofilms exhibit boosted antibiotic resistance, contributing to persistent infections in cystic fibrosis patients [123]. Although Ref. [128] showed that limited oxygen can influence antibiotic resistance in *P. aeruginosa* and reported that the antibiotic was effective only in the oxygenated portion of the biofilm (within 50–90 µm) of the air–biofilm interface. These interconnected factors make biofilms highly resilient, presenting significant challenges in infection control and treatment.

By targeting these resistance mechanisms, we may be able to develop more effective treatments for biofilm-associated infections and address the challenges posed by biofilms in industrial and environmental settings.

## 5. Current and Emerging Antibiofilm Strategies

Biofilms present significant clinical and industrial challenges due to their ability to protect microorganisms from antimicrobial agents, making infections persistent and difficult to eradicate [129]. As a result, research efforts have intensified to develop innovative strategies that target multiple aspects of biofilm formation, maintenance, and dispersal [130]. These approaches primarily focus on two key objectives: preventing biofilm formation and promoting the dispersal of established biofilms [131]. Prevention strategies typically target initial bacterial adhesion through surface engineering or by disrupting crucial bacterial processes such as adhesin expression, EPS synthesis, and quorum sensing signaling [132]. Conversely, dispersal approaches focus on eradicating existing biofilms by degrading the matrix components or triggering bacterial detachment [133]. Current interventions encompass chemical, physical, and biological methods, each offering unique advantages in combating biofilm-associated infections [91,134]. Furthermore, recent advances include exploring diverse agents, from natural extracts to synthetic compounds, often enhanced through novel delivery systems like nanoparticle encapsulation and synergistic drug combinations [135]. While cytotoxicity and in vivo efficacy challenges persist, these emerging strategies represent promising alternatives to conventional antibiotic treatments, offering comprehensive solutions for biofilm control across all developmental stages. Nevertheless, approaches that target multiple aspects of biofilm resistance simultaneously are likely to be more successful than those focusing on a single mechanism [136]. Thus, combination therapies, which involve pairing antibiofilm agents with conventional antibiotics or other compounds, have emerged as a promising strategy to combat biofilm infections by enhancing penetration and efficacy against these complex microbial communities [137].

**Figure 4:**
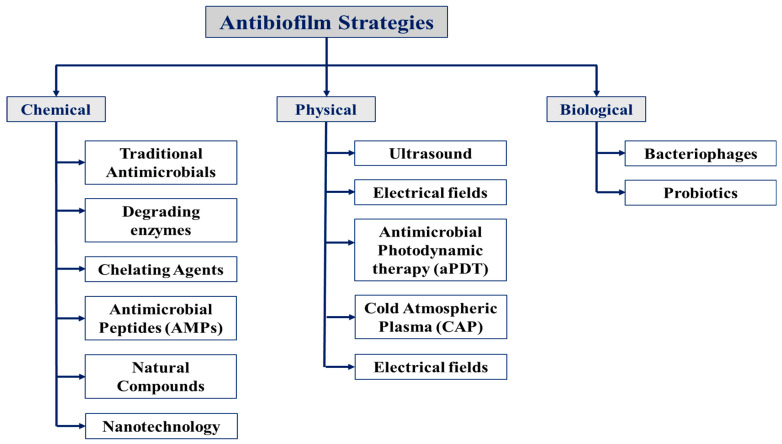
Schematic overview of the antibiofilm strategies discussed in this review. These strategies are categorized into three main approaches: chemical, physical, and biological, each targeting biofilms through distinct mechanisms, including their combinations for enhanced effectiveness.

The various strategies to inhibit or eradicate biofilm formation are illustrated in Figure 4.

### 5.1. Chemical Approaches

Chemical strategies employ a wide range of compounds to inhibit biofilm formation, disrupt established biofilms, or potentiate other therapeutic approaches. These include traditional antimicrobials, degrading enzymes such as DNases and proteases, chelating agents like EDTA, antimicrobial peptides (AMPs), natural compounds (e.g., phenolics and essential oils), and various nanoparticles (metallic, lipid-based, or polymeric). These agents act through multiple mechanisms, including increasing membrane permeability, disrupting cell wall integrity, interfering with quorum-sensing pathways, inhibiting cell division, or inducing biofilm dispersion. Each of these approaches will be discussed in more detail in the corresponding subsections.

#### 5.1.1. Traditional Antimicrobials

Traditional antibiotics remain one of the primary methods for combating biofilm infections, despite their limitations in penetrating these structured bacterial communities. Biofilms exhibit increased resistance to conventional antibiotic treatments, presenting a significant therapeutic challenge [138]. These antimicrobial agents function through five main mechanisms: disrupting bacterial cell wall synthesis (penicillins and cephalosporins), interfering with protein synthesis (tetracyclines, aminoglycosides, macrolides), inhibiting nucleic acid synthesis (rifampicin), compromising cell membrane integrity (polymyxins and colistins, together with antimicrobial peptides (AMPs)), or competitive inhibitors (sulfonamides) [139]. However, several studies have highlighted the limited effectiveness of antibiotics against biofilms. For example, ref. [140] reported that high concentrations of colistin inhibited 20% to 50% of *A. baumannii* biofilms, while ref. [141] found that tobramycin exhibited limited efficacy against *S. aureus* biofilms, with only 10% to 20% inhibition.

Therefore, to overcome these challenges, researchers have explored modified antibiotic strategies. One such strategy includes high-dose antibiotic applications, which involve administering antibiotics at significantly higher concentrations than those typically used for planktonic infections [142]. The reasoning behind higher antibiotic concentrations is to enhance penetration into the dense biofilm matrix and achieve effective bacterial eradication. However, this approach risks increased toxicity and may not eliminate the biofilm [5]. In response, synergistic drug combinations have emerged as a promising approach to enhance biofilm control [137]. For instance, combining antibiotics like tobramycin or ciprofloxacin with agents that disrupt the EPS matrix, such as DNase I or alginate lyase, can significantly improve antibiotic diffusion and reduce biofilm biomass, as demonstrated by ref. [18]. Furthermore, beta-lactams combined with aminoglycosides or fluoroquinolones with rifampicin have been shown to enhance bacterial killing, prevent resistance development, and expand the spectrum of activity. Therefore, several antibiotic combinations are summarized in Table 1. Ref. [143] further demonstrated the strong synergistic effect of fosfomycin, ciprofloxacin, and gentamicin, which resulted in 80–90% biofilm inhibition against *E. coli* and *P. aeruginosa*. Moreover, ciprofloxacin with Ethylenediaminetetraacetic Acid (EDTA) [144] and colistin with carbapenem [145] outperform monotherapies by improving membrane permeability and biofilm disruption (Table 1). Similarly, clarithromycin and vancomycin have exhibited notable efficacy against both planktonic and biofilm-associated bacterial cells, particularly *P. aeruginosa* and *Staphylococcus* species, by targeting alginate, a key EPS component that impairs antibiotic penetration [136]. In another study, ref. [146] showed that tobramycin combined with DNase I degraded eDNA, doubling inhibition rates to achieve 50–60% biofilm reduction. Furthermore, innovative drug delivery systems enhance the effectiveness of traditional antibiotics. For instance, nanoparticles can encapsulate antibiotics and deliver them directly to biofilm sites, thereby increasing local drug concentrations while minimizing systemic toxicity [120]. Similarly, hydrogels enable the controlled release of antimicrobials at infection sites, ensuring prolonged efficacy against biofilm-associated pathogens [147]. Another promising approach involves incorporating antibiotics into biomaterials for medical devices, implants, or wound dressings, facilitating localized and sustained drug release to prevent or delay biofilm formation [148]. Techniques such as coating, impregnation, and encapsulation within biomaterial matrices enhance drug stability and targeted delivery [149]. Continued research into synergistic antibiotic therapies, biofilm-disrupting agents, and nanotechnology-based drug delivery systems will be essential for overcoming the therapeutic challenges posed by biofilm-associated infections.

#### 5.1.2. Degrading Enzymes

The enzymatic degradation of biofilms represents a highly selective and effective strategy for biofilm disruption, as it relies on the chemical structure of enzymes and their specific target components [150]. By breaking down key structural components of the biofilm matrix, these enzymes primarily target EPS and bacterial cell walls, weakening biofilm integrity and enhancing the penetration of antibiotics and immune cells while promoting biofilm dispersal [87,151]. One major group of matrix-degrading enzymes includes glycosidases, which hydrolyze specific polysaccharides in the EPS [152]. For example, dextranase breaks down dextran in oral biofilms, α-amylase degrades starch-like polysaccharides, and alginate lyase specifically targets alginate in *P. aeruginosa* biofilms, and hyaluronidase hydrolyzes hyaluronic acid [39,153]. Additionally, β-N-acetylglucosaminidases target N-acetylglucosamine in fungal or chitin-containing bacterial biofilms, while Dispersin B, a glycoside hydrolase, weakens biofilm matrices by hydrolyzing glycosidic bonds within EPS polysaccharides, including the PNAG polymers that facilitate bacterial aggregation [23,154]. As presented in Table 1, the effectiveness of Dispersin B has been demonstrated against *S. epidermidis* biofilms, where 0.13 μg to 3.20 μg of protein per sample exhibited high antibiofilm activity [155]. Another category of biofilm-degrading enzymes includes proteases, such as trypsin and proteinase K, which target proteins within the EPS. By cleaving peptide bonds, these enzymes further destabilize the biofilm structure [156]. Furthermore, deoxyribonucleases (DNases) degrade eDNA, another crucial EPS component, by hydrolyzing phosphodiester bonds [157,158]. For example, DNase I has been shown to significantly reduce *P. aeruginosa* and *S. aureus* biofilm formation at a concentration of 1 mg/mL, as demonstrated by Ref. [159] (Table 1). Moreover, ref. [152] reported that a combination of DNase I and Dispersin B successfully inhibited staphylococcal skin colonization and removed pre-attached *S. aureus* cells. Beyond single-enzyme applications, innovative approaches that combine enzymes with other antibiofilm technologies have yielded promising results. Recent studies by ref. [160] demonstrated that a bioconjugate of α-amylase and silver nanoparticles (AgNP), which integrates a degrading enzyme with nanotechnology, achieved 78% and 73% inhibition of *K. pneumoniae* and *S. aureus* biofilms, respectively, by hydrolyzing polysaccharides and disrupting cell membranes at a concentration of 800 μg/mL. Apart from targeting EPS components, some enzymes act directly on bacterial cell walls. Lysozyme, for example, hydrolyzes peptidoglycan in Gram-positive bacteria, while peptidoglycan hydrolases such as lysostaphin specifically degrade *S. aureus* cell walls, leading to bacterial lysis within the biofilm [93]. Similarly, ref. [161] illustrated that combining oxytetracycline with lysozyme and EDTA resulted in 63% biofilm eradication, with 2.8 mg/mL of the antibiotic and 26 mg/mL of lysozyme mixed with EDTA, demonstrating a strong synergistic effect. Recent studies by [29] have revealed that natural biofilm dispersion of *S. epidermidis* is closely tied to compositional changes in the matrix, such as a reduction in polysaccharides and an increase in proteins, making the biofilm more susceptible to enzymatic attack. In particular, the presence of PNAG positively correlated with enzymatic susceptibility, highlighting the potential of targeting matrix components like polysaccharides and proteins using glycoside hydrolases and proteases [29]. These findings underscore the importance of understanding matrix dynamics during dispersion to optimize enzyme-based biofilm disruption strategies. These enzymatic strategies offer significant advantages, including high specificity, synergy with other antibiofilm agents, and a lower resistance development risk than traditional antibiotics. Despite their potential, the widespread application of matrix-degrading enzymes is limited by cost, handling complexity, and industrial accessibility [162]. Challenges related to enzyme stability, efficient delivery, and large-scale production must be addressed to enhance their feasibility in clinical and industrial settings. However, with continued advancements in enzyme engineering, nanotechnology-based delivery systems, and synergistic combinations, enzymatic degradation remains a promising avenue for biofilm control and eradication.

#### 5.1.3. Chelating Agents

Chelating agents represent another strategy in combating biofilms by targeting the crucial role of metal ions in biofilm formation, stability, and bacterial metabolism. Metal ions (such as Zn^2+^, Mg^2+^, Fe^2+^, and Ca^2+^) serve as essential cofactors for bacterial enzymes involved in vital metabolic pathways, contribute to the structural integrity of the EPS matrix through cross-linking its components, mediate bacterial adhesion to surfaces, and are even implicated in quorum sensing and oxidative stress response [129,163]. Chelating agents, such as EDTA and lactoferrin, disrupt these processes by binding to metal ions, thereby removing them from the biofilm environment [164]. EDTA, a widely used chelator, can also damage the cell wall, subsequently disrupting the biofilms via sequestering zinc, magnesium, iron, and calcium [165]. EDTA is generally considered safe for use in prescription medicine and in small amounts as a food preservative [166]. Moreover, it is often combined with antibiotics for synergistic effects. Ref. [167] has demonstrated that lysozyme combined with EDTA can destabilize the biofilm and has been used for the disruption of *E. coli*, *S. aureus*, and *P. aeruginosa* biofilms. Furthermore, lactoferrin, an iron-binding protein, deprives bacteria of iron while simultaneously disrupting the EPS matrix and enhancing antibiotic activity [66]. Hence, this leads to nutrient deprivation, weakened biofilm structure due to disrupted metal ion cross-linking, and increased antibiotic penetration [168]. This disruption also enhances the susceptibility of biofilm bacteria to other antibiofilm agents and host immune responses, sometimes even interfering with QS [168]. Similarly, chitosan is a natural polymer used in numerous applications in the biomedical field because of its biodegradability, bioadhesive properties, and bioactivity [13]. It is known to disrupt negatively charged cell membranes due to its cationic nature [169]. Thus, we can combat the bacteria in the early stages of biofilm formation by employing such chemicals. Despite their advantages, such as broad-spectrum activity, synergistic potential with antibiotics, and relatively low toxicity, chelating agents face challenges [165]. Their non-specificity can potentially affect host tissues, and their efficacy is often greater when combined with other antibiofilm approaches [151]. Effective delivery and maintaining their stability at the target site also present challenges. Nevertheless, by targeting the fundamental role of metal ions and cell wall integrity, these agents offer a distinct and promising approach to biofilm control.

#### 5.1.4. Antimicrobial Peptides (AMPs)

Antimicrobial peptides (AMPs) have emerged as a promising alternative to traditional antibiotics for treating biofilm-associated infections. AMPs are small molecules produced as part of the innate immune response in various organisms, including bacteria, fungi, plants, and animals [170,171]. Typically composed of 10 to 50 amino acids, AMPs can be classified based on their structural characteristics as either α-helical, β-stranded, looped, β-hairpin, or extended forms [172]. Moreover, these peptides exhibit broad-spectrum antimicrobial activity and are promising candidates for new anti-biofilm strategies [13]. The cationic amphipathic structure of these AMPs allows them to interact with negatively charged bacterial membranes, leading to cell lysis or invasion [173]. However, AMPs employ several non-membranolytic mechanisms of action beyond membrane disruption [174]. These include inhibition of cell wall synthesis, interference with nucleic acid synthesis, modulation of immune responses, and disruption of essential metabolic processes within microorganisms [174]. Notably, research indicates that AMPs can prevent biofilm formation by disrupting bacterial signaling pathways and reducing the expression of genes necessary for biofilm development [175]. Additionally, they can target established biofilms by acting on bacterial membrane potential, highlighting their potential as therapeutic agents in combating biofilm-associated infections [173]. While bacterial resistance to AMPs is less common than with traditional antibiotics, it remains a concern that warrants attention [176]. Furthermore, the effectiveness of AMPs can be enhanced when combined with other agents, such as traditional antibiotics or compounds that disrupt the extracellular matrix of biofilms. These combinations can effectively address the heterogeneous nature of biofilm communities by targeting cells in various metabolic [177]. Despite these advantages, several limitations hinder the clinical use of AMPs. One significant challenge is their short half-life; AMPs are rapidly degraded by proteolytic enzymes, which reduces their effectiveness against biofilms [178]. Additionally, some AMPs can exhibit toxicity to mammalian cells, raising safety concerns [135]. Moreover, their efficacy may vary among different bacterial species and biofilm types, complicating the development of universally effective peptides [179]. Many AMPs also suffer from poor pharmacokinetics, which affects absorption and distribution within the body. However, AMPs are not only naturally occurring molecules but can also be synthesized to enhance their efficacy and stability against biofilms [180]. Synthetic AMPs offer the advantage of being designed to target specific bacterial structures or mechanisms, providing a tailored approach to combat biofilm-associated infections [171]. Several examples highlighting these diverse mechanisms are reported in Table 1. Nisin reduces *S. aureus* adhesion and alters cell and surface hydrophobicity (Table 1) [181]. Ref. [171] showed that P30, a synthetic AMP, reduced 2.62 log CFU/mL of *A. baumannii* biofilm by the formation of transmembrane pores, causing the loss of bacterial viability. Lin-SB056-1 targets extracellular polysaccharide components of *S. epidermidis*, leading to a significant reduction in biofilm biomass [137]. Ref. [182] showed that the combination of 1Tb and protease effectively disrupts membranes and degrades proteins in *Enterococcus faecalis*, achieving a 70–80% reduction in biofilm biomass (MIC: 0.5–1 μg/mL with protease, IC50: 10 μg/mL for protease). Additionally, the combination of AMP38 and imipenem demonstrated synergistic killing and effective biofilm eradication against multidrug-resistant *P. aeruginosa* [183]. The combination of Ana-9 and oxacillin sodium monohydrate significantly inhibits biofilms of methicillin-resistant *S. aureus* and *S. epidermidis*, with biofilm inhibition ranging from 75–90% [184]. Moreover, combining the semi-synthetic peptide Lin-SB056-1 and EDTA shows potent antibiofilm activity against both mucoid and non-mucoid *P. aeruginosa*, including CF isolates, by disrupting bacterial membranes and destabilizing the biofilm matrix; it achieves rapid bactericidal action and significantly reduces biofilm biomass and formation [185]. Furthermore, despite over 3000 AMPs having been discovered, only a few are Food and Drug Administration (FDA)-approved, highlighting the need for further research to address issues like cytotoxicity and side effects [186]. To overcome these challenges, improved formulation and delivery methods are essential for enhancing the stability and solubility of AMPs [187]; innovative approaches such as nanocarrier systems are being explored to tackle these issues effectively [188]. Overall, addressing these limitations is essential for the successful integration of AMPs into therapeutic strategies against biofilm-associated infections, and ongoing advances in peptide engineering, formulation techniques, and combination therapies hold promise for overcoming these challenges and maximizing their clinical potential.

#### 5.1.5. Natural Compounds

Natural compounds represent a significant category of antibiofilm agents, derived from various organisms and showcasing diverse mechanisms of action. These compounds are extracted from a wide range of natural sources, including plants such as essential oils, polyphenols, plant extracts, microorganisms like bacteriocins, and marine organisms such as algae and sponges [189]. Their natural origin contributes to their bioactivity and therapeutic potential against biofilm-forming pathogens [135]. The mechanisms by which natural compounds combat biofilms are diverse and multifaceted. One key mechanism is the inhibition of EPS production, which is critical for biofilm formation [190]. By interfering with EPS synthesis, these compounds can prevent the establishment of new biofilms or weaken existing ones [21]. Another important mechanism is QS inhibition, where natural compounds disrupt bacterial communication systems that are essential for coordinating biofilm development [191]. Additionally, many natural compounds can disrupt bacterial membranes, leading to cell lysis and bacterial death, which is particularly effective against mature biofilms [187]. Furthermore, some compounds can interfere with bacterial adhesion to surfaces, blocking the initial stages of biofilm formation and reducing the risk of biofilm-related infections [51]. Numerous natural compounds have been identified for their anti-biofilm properties. For example, essential oils such as tea tree oil, cinnamon oil, clove oil, and oregano oil have demonstrated effectiveness against various pathogens [192]. Similarly, plant extracts like green tea extract, cranberry extract, and pomegranate extract have shown inhibitory effects on biofilm formation [193]. Other notable examples include alkaloids such as berberine and sanguinarine, flavonoids like quercetin and naringenin, and terpenoids such as farnesol and limonene [81]. These compounds exhibit a wide range of activities that target different stages of biofilm formation and maintenance. Recent research has highlighted the efficacy of natural compounds such as curcumin, cinnamaldehyde, eugenol, and thymol in both preventing biofilm formation and eradicating mature biofilms [193,194]. For instance, curcumin has been shown to prevent bacterial adhesion by interacting with enzymes like sortase A, which are essential for attachment. Moreover, combining natural compounds with conventional antibiotics has been found to enhance therapeutic outcomes by increasing potency while reducing toxicity and the likelihood of resistance development [195]. In Table 1, curcumin is shown to inhibit pellicle formation, pili motility, and ring biofilm formation in *A. baumannii* and *Candida albicans*. Molecular docking analysis revealed that curcumin interacts with the biofilm response regulator BfmR. At a concentration of 100 mg/mL, curcumin reduces biofilm production by 93% [195]. Clove essential oil (CEO) and oregano essential oil (OEO), containing eugenol and carvacrol, respectively, suppressed the metabolic activity and extracellular polysaccharide production in *Salmonella derby*. Notably, at 1/8 MIC, CEO inhibited biofilm formation by 90.29%, while OEO showed 48.79% inhibition at 0.8 mg/mL and 0.2 mg/mL, respectively [196]. Despite their potential benefits, natural compounds face several limitations that can restrain their clinical application. A major challenge is the variability in efficacy, as their effectiveness can differ significantly among various bacterial species and biofilm types [197]. Additionally, some natural compounds may exhibit toxicity, thus raising safety concerns [194]. Many also suffer from poor solubility and stability, which can limit their bioavailability and effectiveness in vivo [198]. Furthermore, there is a risk of bacterial resistance developing against certain natural compounds over time, although this risk is generally lower compared to synthetic antibiotics [120]. Finally, while many natural compounds have shown promise in laboratory settings, there is often a lack of comprehensive clinical studies to validate their efficacy in real-world applications. Continued research into these agents is essential for developing effective strategies that maximize their benefits while minimizing adverse effects and resistance development.

#### 5.1.6. Nanotechnology

Nanotechnology provides promising tools for combating biofilms, especially using nanoparticles (NPs), which have been extensively researched for their antibacterial and antibiofilm properties. Different types of NPs have been designed as antimicrobial and antibiofilm agents, such as organic, inorganic, metal, and green NPs [199]. Among these, silver nanoparticles (AgNPs) are particularly noteworthy due to their inherent bactericidal abilities and effectiveness against biofilm-forming bacteria such as *P. aeruginosa*, *E. coli*, *S. aureus*, and *S. epidermidis* [200]. AgNPs exhibit their antibiofilm activity through multiple mechanisms, including disruption of bacterial membranes, generation of reactive oxygen species (ROS), inhibition of bacterial adhesion, and interference with the synthesis of EPS [201]. By adhering to bacterial cell walls, AgNPs compromise membrane integrity, causing structural damage and leakage of cellular contents. Furthermore, they induce oxidative stress, which further weakens bacterial cells and prevents biofilm formation [202]. Their ability to inhibit bacterial adhesion prevents the initial establishment of biofilms, while interfering with EPS synthesis disrupts the matrix that holds biofilm structures together [203]. Additionally, synthesizing AgNPs using natural extracts enhances both their antibacterial properties and eco-friendliness, positioning them as a promising alternative to traditional chemical synthesis methods [51]. Their compatibility with antibiotics further increases treatment efficacy against antibiotic-resistant bacteria [204]. In addition to their direct antimicrobial effects, nanoparticles also serve as drug delivery vehicles, enhancing the localized administration of antimicrobials such as vancomycin, ciprofloxacin, and farnesol [205]. By encapsulating antibiotics within nanoparticle carriers, these systems improve drug penetration into biofilms, safeguard drugs from degradation, minimize systemic side effects, and combat antibiotic resistance by providing more targeted and sustained antimicrobial activity [206]. Table 1 summarizes several nanoparticles, including tryptone-stabilized silver nanoparticles (Ts-AgNPs) effectively distort the matrix and mature biofilms of *K. pneumoniae* and *P. aeruginosa*, and they also attenuate quorum-sensing-induced virulence factor production [207]. Ts-AgNPs showed up to 93% biofilm inhibition and 97% eradication, with MIC50 values of 1.7 μg/mL for *K. pneumoniae* and 2.7 μg/mL for *P. aeruginosa*. Furthermore, AgNPs synthesized using *Terminalia chebulic* fruit extract increased membrane permeability and caused cell death, achieving 80% inhibition in *P. aeruginosa*, 75% in *E. coli*, and 70% in *S. aureus* biofilms at a concentration of 100 μg/mL [201]. A recent study conducted by ref. [208] demonstrated that gold nanoparticles (AuNPs) combined with ampicillin enhanced antibiotic efficacy, disrupting biofilms by 50–60% at effective concentrations of 10–50 μg/mL for both AuNPs and ampicillin. Interestingly, recent advancements in nanotechnology have led to the development of specialized nanoparticles designed to improve biofilm disruption and antimicrobial effectiveness further [209]. Although nitric oxide-containing silica nanoparticles release antimicrobial nitric oxide to kill bacteria within biofilms, superparamagnetic iron oxide nanoparticles disrupt EPS matrices and bacterial cell membranes through effects induced by magnetic fields [210]. Liposomal nanoparticles represent another promising avenue for drug delivery, as they can fuse with bacterial membranes to release antimicrobials intracellularly. Moreover, stimuli-responsive nanoparticles, triggered by specific environmental factors such as pH or bacterial enzymes, enable controlled drug release and targeted antimicrobial action [210]. These emerging nanotechnologies hold significant potential for creating more effective biofilm prevention and removal strategies. Despite their promise, additional research is essential to enhance the in vivo efficacy and biocompatibility of nanoparticle-based treatments, as well as to fully understand their cytotoxicity, metabolism, and long-term environmental impact. Furthermore, developing cost-effective and scalable manufacturing processes remains a key challenge in translating nanoparticle-based antibiofilm strategies into widespread clinical and industrial applications. By addressing these limitations, nanotechnology has the potential to transform biofilm prevention and control, providing highly effective and innovative solutions to combat bacterial infections.

The following table summarizes various chemical strategies and agents employed for antibiofilm activity, outlining their mechanisms of action, efficacy, and concentrations used in different bacterial species. It highlights recent advances in the field of biofilm disruption.

**Table 1:** Selected chemical antibiofilm agents, their mechanisms of action, and their efficacy.

Strategy	Antibiofilm Agent	Target Bacteria	Mechanism of Action	Efficacy	MIC/MBC/ IC50	Reference
**Antibiotic**	Ciprofloxacin	*P. aeruginosa*	Inhibits DNA replication	Moderate inhibition (30–40%)	MIC: 1–2 μg/mL	[211]
**Antibiotic + chelating agent**	Ciprofloxacin + EDTA	*P. aeruginosa*	Enhances membrane permeability	High inhibition (70–80%)	MIC: 0.5–1 μg/mL (with EDTA IC50: 10 μM)	[144]
**Antibiotic**	Colistin	*A. baumannii*	Disrupts bacterial membranes	20–50% inhibition	MIC: 2–4 μg/mL	[140]
**Antibiotics combination**	Colistin + Carbapenem	*A. baumannii*	Enhanced disruption	60–80% inhibition	MIC: 1–2 μg/mL (with Carbapenem, IC50: 1 μg/mL)	[145]
**Antibiotics combination**	Fosfomycin + Ciprofloxacin + Gentamicin	*E. coli,* *P. aeruginosa*	Synergistic action: Fosfomycin (cell wall synthesis inhibition), Ciprofloxacin (DNA replication inhibition), Gentamicin (protein synthesis inhibition)	80–90% biofilm inhibition	MICs reduced 2–8×	[143]
**Antibiotics combinations**	Clarithromycin/Vancomycin + Imipenem	*P. aeruginosa* and *Staphylococcus* species	Breaks down EPS components	N/A	N/A	[18]
**Antibiotic**	Tobramycin	*S. aureus*	Interference with protein synthesis	Limited inhibition (10–20%)	MIC: 2–4 μg/mL,	[141]
**Antibiotic with degrading enzyme**	Tobramycin + DNase I	*S. aureus*	DNA degradation in biofilms	Enhanced disruption (50–60% inhibition)	MIC: 1–2 μg/mL (with DNase I: IC50: 5 μg/mL)	[146]
**Degrading enzymes**	DNase I	*P. aeruginosa* and *S. aureus*	DNA degradation in biofilms; reducing structural integrity	Reduced biofilm formation relative to uncoated surfaces	DNase I concentration: 1 mg/mL	[159]
**Degrading enzymes**	Dispersin B	*S. epidermidis*	Hydrolyzes PNAG, disrupting EPS	High activity	0.13–3.20 μg/sample	[155]
**Degrading enzymes + nanotechnology**	α-amylase-AgNPs	*K. pneumoniae* and *S. aureus*	EPS and membrane disruption	78% and 73% of biofilm inhibition for *K. pneumoniae* and *S. aureus*, respectively	800 μg/mL	[160]
**Degrading enzyme + chelating agent**	Lysozyme + EDTA	*S. epidermidis*	EDTA enhances membrane permeability for lysozyme	Eradication reached a peak of 74%	IC50: 80 mg/mL of lysozyme-EDTA	[161]
**Antibiotic**	Oxytetracycline hydrochloride	*S. epidermidis*	Oxytetracycline effectively reduces biofilm biomass	Eradication reached a maximum of 43%	2.8 mg/mL oxytetracycline hydrochloride	
**Antibiotic + Degrading enzyme + chelating agent**	Oxytetracycline hydrochloride + lysozyme + EDTA	*S. epidermidis*	Synergistic effect	Eradication reached a maximum of 63%	2.8 mg/mL antibiotic with 26 mg/mL of lysozyme-EDTA.	
**AMPs**	Nisin	*S. aureus*	Inhibits cell wall synthesis, disrupting cell membrane integrity	Reduced cell adhesion	0.4 μM	[181]
**AMPs**	P30	*A. baumannii CRAB KPD 205*	Transmembrane pore formation, causing the loss of bacterial viability.	Reduction levels of 2.62 log CFU/mL	N/A	[212]
**AMPs**	Lin-SB056-1	*S. epidermidis*	Targets ESP components	Significant reduction in biofilm biomass	N/A	[137]
**Amps + degrading enzyme**	1Tb + Protease	*E. faecalis*	Membrane disruption with protein degradation	Significant reduction in biomass (70–80%)	MIC: 0.5–1 μg/mL (with Protease, IC50: 10 μg/mL)	[182]
**AMPs + antibiotic**	AMP38 + Imipenem	MDR *P. aeruginosa*	AMP38 enhances imipenem uptake by disrupting the outer membrane	Synergistic killing and biofilm eradication	MBEC (combination): 62.5 µg/mL	[183]
**AMPS + antibiotic**	Ana-9 + Oxacillin sodium monohydrate	MRSA and MRSE	Synergistic inhibition	75–90% inhibition	MBEC: oxacillin: 2048–8192 µg/mL and nisin: 2048–4096 µg/mL	[184]
**AMP + chelating agent**	Lin-SB056-1 + EDTA	*P. aeruginosa*	Peptide disrupts membranes; EDTA destabilizes biofilm matrix	Rapid killing; up to 80% biofilm reduction	25 µg/mL + 0.6–1.25 mM EDTA	[185]
**Natural compounds**	Curcumin	*A. baumannii*, *C. albicans*	Disrupts biofilm structure and mobility by targeting the BfmR regulator.	Reduces biofilm production by 93%	100 mg/mL	[195]
**Natural compounds**	Clove essential oil and Oregano essential oil	*S. derby*	Suppressing metabolic activity and EPS production	49–90% inhibition	0.8 mg/mL and 0.2 mg/mL, respectively	[196]
**Nanotechnology**	Tryptone stabilized AgNPs (Ts-AgNPs)	*K. pneumoniae* and *P. aeruginosa*	Matrix distortion, QS inhibition	Up to 97% biofilm inhibition and eradication	MIC50: 1.7 μg/mL and 2.7 μg/mL for *K. pneumoniae* and *P. aeruginosa,* respectively.	[207]
**Nanotechnology**	AgNPs (plant derived)	*P. aeruginosa*, *E. coli*, *S. aureus*	Increased membrane permeability and subsequent cell death	*P. aeruginosa*: 80% inhibition *E. coli:* 75% inhibition *S. aureus:* 70% inhibition	100 μg/mL	[201]
**Nanotechnology + antibiotic**	AuNPs + Ampicillin	*E. coli*	Enhanced antibiotic delivery and efficacy	50–60% inhibition	10–50 μg/mL (AuNPs), 10–50 μg/mL (Ampicillin)	[208]

This review provides a comprehensive understanding of biofilm eradication by examining a range of strategies. Initially, we explore chemical methods that have long been foundational in antibiofilm interventions. However, their effectiveness is often hindered by challenges such as resistance development and incomplete biofilm eradication. To address these limitations, researchers have increasingly turned to physical approaches to disrupt biofilm structures and enhance antimicrobial penetration.

### 5.2. Physical Approaches

Physical antibiofilm methods are based on various approaches to prevent and control biofilm formation, offering eco-friendly alternatives to traditional antimicrobial treatments. These strategies include the application of ultrasound, electric or magnetic fields, photodynamic therapy, and many others. As an example, ultrasound can disrupt biofilm structures and enhance the penetration of antimicrobial agents, while electric fields can promote the detachment of bacteria from surfaces [213]. The remarkable microbiocidal properties of these physical methods make them promising tools for combating biofilms in various settings, including medical devices and food safety applications. By leveraging these innovative approaches, researchers aim to develop effective strategies that minimize reliance on chemical agents while addressing the challenges posed by biofilm-associated infections.

#### 5.2.1. Ultrasound

Ultrasound is increasingly recognized as an effective physical strategy for preventing and controlling biofilm formation. This technique utilizes high-frequency sound waves to disrupt biofilm structures. It is also employed as part of a hurdle treatment strategy to increase bacterial membrane permeability and enhance the effectiveness of antimicrobial agents [214]. Ultrasound’s antibiofilm effects are primarily mediated by mechanical forces that disrupt EPS structures and by cavitation phenomena that damage biofilm architecture. Thus, cavitation occurs when rapid pressure changes create and collapse microbubbles, generating shock waves and high-speed microjets that mechanically disrupt surrounding structures [215]. In biofilms, this results in the breakdown of the EPS matrix, the physical dislodgement of bacterial cells, and the formation of microchannels that enhance antimicrobial penetration [216]. Additionally, studies in plant tissues suggest that cavitation also induces localized heating, softening biological matrices, and further facilitating the diffusion of solutes, a mechanism that may similarly enhance biofilm disruption [216]. High-intensity ultrasound (HIUS) at 20 kHz and 120 W, illustrated in Table 2, causes the formation and collapse of microbubbles, generating localized high-pressure and high-temperature conditions that disrupt the *S. aureus* biofilm matrix, leading to a 55% reduction in biofilm viability after just 1 min of exposure [217]. Other studies have demonstrated that ultrasound, particularly when combined with microbubbles, enhances the activity of antibiotics like vancomycin against biofilms formed by *S. epidermidis*, leading to significant biomass reduction [214]. Additionally, ultrasound disrupts biofilm formation at early stages by interfering with bacterial adhesion and signaling pathways, including autoinducer gradients and microstreaming effects. Moreover, the effectiveness of ultrasound depends on factors such as frequency, intensity, and exposure duration [218]. Low-frequency, high-intensity ultrasound has shown strong bactericidal properties, and its combination with antimicrobial agents further improves treatment outcomes [215]. Similarly, as presented in Table 2, combining low-frequency ultrasound with conventional antibiotics markedly improves treatment outcomes by disrupting the biofilm architecture and enhancing drug penetration, leading to significantly greater bacterial reduction compared to antibiotics alone [219]. However, efficacy can vary depending on bacterial species and biofilm type, and some bacteria may develop adaptive responses, such as increased production of cyclic-di-GMP, which stabilizes biofilms [220]. There is also a risk of thermal damage to surrounding tissues if treatment parameters are not carefully controlled. Furthermore, while ultrasound enhances antibiotic penetration, it often requires combination therapies, which can increase complexity and cost [39]. Ref. [174] reported that combining ultrasound-targeted microbubble destruction (UTMD) with human β-defensin-3 (HBD-3) significantly enhances the inhibition of biofilm-associated gene expression in MRSA and MRSE, resulting in reduced biofilm density and viable bacterial counts in vivo, as previously demonstrated [221] (Table 2). Despite these challenges, ultrasound remains a promising non-invasive approach to combating biofilm-associated infections, offering a valuable complement to traditional chemical and biological treatments. Ongoing research aims to optimize its application in clinical settings by refining treatment parameters, minimizing adverse effects, and improving its standalone efficacy.

#### 5.2.2. Electrical Fields and Pulsed Electrical Fields (PEF)

Electrical fields are emerging as a promising physical strategy for preventing and disrupting biofilm formation. This approach is based on the application of weak direct current (DC) electric fields to alter the behavior of bacteria and enhance the efficacy of antimicrobial treatments [132]. One of the primary mechanisms by which electrical fields exert their antibiofilm effects is through electroporation, where electrical stimulation creates pores in bacterial cell membranes. This process can lead to cell damage and increased permeability, making bacteria more susceptible to antimicrobial agents [154]. Additionally, electrical fields can induce changes in the ambient environment, such as altering pH levels and generating ROS through electrolysis, which further inhibits bacterial growth and survival [213]. Research has demonstrated that combining electrical fields with antibiotics enhances antimicrobial effectiveness, a phenomenon known as the bioelectric effect, where low doses of antibiotics paired with electric fields disrupt mature biofilms [154]. For instance, studies have shown that using electric fields can significantly reduce the survival rates of bacteria within biofilms, including *P. aeruginosa* and *A. baumannii* [222]. Table 2 highlights the effectiveness of low-amperage DC in disrupting biofilm structures and eliminating sessile cells through electrical stimulation. For instance, applying 500 μA DC over four days successfully eradicated *S. aureus* and *S. epidermidis* biofilms while reducing *P. aeruginosa* and *E. coli* biofilms by 5.2–5.5 log CFU/cm^2^ [213]. Platinum electrodes delivering DC exhibit a dose- and time-dependent reduction in viable biofilm cells, with significant decreases observed at 200 μA DC for 24 h (1.4–2.1 log CFU/cm^2^) and complete eradication achieved after four days at 500 μA DC. Additionally, Pulsed Electric Fields (PEFs) emerge as an alternative strategy for biofilm disruption, applying short, high-voltage pulses to permeabilize bacterial membranes [223]. A study investigated the effects of extremely low-frequency pulsed electric fields (ELF-EF) on *K. pneumoniae*. The researchers found that exposure to 0.8 Hz for 60 min significantly inhibited bacterial growth and biofilm formation, and increased antibiotic susceptibility [223]. While initially explored in the food industry, PEF is increasingly studied for biomedical applications due to its non-thermal, localized effects that enhance bacterial inactivation and antimicrobial penetration [224]. Despite their promising potential, both electrical field and PEF approaches face challenges. Variability in bacterial species responses and differences in biofilm structure can affect treatment outcomes. Furthermore, careful optimization of parameters, such as voltage intensity, frequency, pulse duration, and total treatment time, is critical to maximize antibiofilm effects while minimizing damage to surrounding tissues [225]. Continued research is necessary to standardize treatment protocols and fully understand the mechanistic effects of both continuous and pulsed electrical strategies on biofilm communities.

#### 5.2.3. Antimicrobial Photodynamic Therapy

Antimicrobial photodynamic therapy (aPDT) is an innovative physical strategy for combating biofilms, leveraging the synergistic effects of light, photosensitizers (PS), and molecular oxygen to generate ROS that effectively disrupt biofilm structures [190]. In aPDT, non-toxic photosensitizers are activated by harmless visible light at specific wavelengths, leading to the production of ROS that oxidize various cellular components, including proteins, lipids, and nucleic acids within the biofilm matrix [226]. This multi-targeted approach allows the penetration and damage of not only the biofilm surface but also the bacterial cells embedded within EPS, making it effective against both planktonic and biofilm-associated bacteria. This strategy has demonstrated effectiveness against drug-resistant bacteria, including both Gram-positive and Gram-negative strains, and has shown promise in eliminating *S. mutans* biofilms associated with oral infections [226]. Furthermore, recent studies have demonstrated that aPDT can significantly reduce biofilm viability in various pathogens, including *S. aureus* and *P. aeruginosa* [227]. Hence, in Table 2, Ref. [228] demonstrates that Rose Bengal-mediated aPDT effectively inactivates MRSA and partially reduces *P. aeruginosa* biofilms through ROS-induced oxidative damage. The therapy’s effectiveness is enhanced when combined with other treatments, such as pulsed PEF, which increases the permeability of the biofilm matrix and facilitates greater penetration of the photosensitizer [229]. Additionally, aPDT has shown minimal development of resistance among bacteria, making it a promising alternative to traditional antimicrobial therapies [133,230]. However, the limited penetration depth of light in biological tissues can restrict the effectiveness of aPDT in deeper or thicker biofilms [149]. However, a recent study demonstrates that a porphyrin nano emulsion overcomes common delivery limitations of photosensitizers, enabling deep penetration into biofilms and effective inactivation of bacterial cells, achieving high reductions (up to 6 log_10_) [231]. Furthermore, the efficacy of photosensitizers can vary based on their binding affinity to bacterial cells and their ability to generate ROS upon light activation. Additionally, optimizing parameters such as light intensity, exposure duration, and photosensitizer concentration is crucial for achieving desired outcomes without causing damage to surrounding healthy tissues [232]. There are also concerns regarding potential phototoxicity to host cells, particularly in sensitive areas. Interestingly, addressing these limitations will be essential for enhancing the clinical applicability and effectiveness of this therapy.

#### 5.2.4. Cold Atmospheric Plasma

Cold atmospheric plasma (CAP) has recently emerged as a powerful physical strategy for biofilm control by generating reactive oxygen and nitrogen species (RONS), UV radiation, and charged particles under non-thermal conditions, thereby disrupting biofilm structures and inactivating embedded bacteria [233]. CAP treatment has been shown to reduce viable cell counts and impair membrane integrity in established biofilms of both Gram-positive and Gram-negative bacteria [234]. Multiple studies demonstrate its effectiveness, with Ref. [235] showing up to 3.08 log reduction in bacterial biofilms, and treatments as short as 60 s are capable of significantly reducing bacterial populations. In a study on *P. aeruginosa* biofilms, sub-lethal CAP pre-treatment dramatically enhanced the efficacy of antibiotics, lowering minimum biofilm eradication concentrations by up to 512-fold for ciprofloxacin/gentamicin and 256-fold for tobramycin (Table 2) [236]. In the case of *S. aureus* and MRSA, direct CAP exposure for up to 180 s achieved up to a ~5.2 log_10_ reduction in viable biofilm bacteria on surfaces, associated with increased intracellular reactive species and damage to cell membranes [237]. CAP array inactivates biofilms on environmental and contaminated surfaces by generating reactive species that damage the EPS matrix and bacterial cells. A 60-s treatment achieved over 99% bacterial reduction, including a 250-fold decrease in 2-day *P. fluorescens* biofilms on steel, demonstrating its fast, effective, and broad-spectrum antibiofilm potential [238]. Mechanistically, CAP exerts oxidative stress-mediated damage to proteins, lipids, and nucleic acids, disrupts cell membrane integrity and increases permeability, and may inactivate biofilms more rapidly than conventional antibiotics, without promoting resistance upon repeated exposure [234]. Because of these attributes, non-thermal, multi-targeted, and effective even against resistant strains, CAP represents a promising addition to the anti-biofilm toolbox, particularly for surface disinfection, medical decontamination, and as an adjuvant to antibiotics.

#### 5.2.5. Micro/Nanomotors

Motors represent an emerging class of physical antibiofilm strategies that actively disrupt biofilm structures through mechanical forces. Recent studies have explored the use of micro- and nanoscale rotary and linear motors capable of penetrating and dismantling biofilm matrices [239]. For example, magnetically driven microbots functionalized with enzymes have been shown to physically break down extracellular polymeric substance (EPS) components while delivering antibacterial agents deep into biofilms, significantly enhancing eradication efficacy [240]. Light-activated synthetic molecular motors have also demonstrated the ability to disrupt biofilms by generating localized mechanical agitation, thereby increasing biofilm permeability and susceptibility to antibiotics [241]. Additionally, ultrasonic micro-motors have been employed to induce shear forces that impair biofilm integrity without damaging surrounding tissues, offering a non-invasive treatment modality [242]. Recent studies, presented in Table 2**,** have demonstrated the potential of micro- and nanomotors as effective physical antibiofilm agents. For example, Janus Pt-mesoporous silica nanomotors functionalized with ficin and loaded with vancomycin have been shown to penetrate *S. aureus* biofilms through self-propulsion, hydrolyze the EPS matrix via ficin, and deliver antibiotics locally, achieving approximately 82% biofilm disruption and 96% bacterial killing [243]. The key advantages include autonomous motion, enhanced penetration of biofilm barriers, and multi-modal treatment approaches [239]. In addition, light- and catalysis-driven cascade nanomotors have demonstrated mechanical penetration of MRSA and mixed biofilms, ROS generation, and enhanced drug delivery, resulting in 91% biofilm degradation in vitro and over 94% bacterial killing in vivo [244]. These motors overcome traditional antibiotic limitations by combining mechanical disruption, precise targeting, and synergistic antimicrobial mechanisms. However, Ref. [244] notes that clinical translation still requires addressing regulatory and biosafety challenges. However, these strategies face significant challenges, including dependence on external energy sources or chemical fuels (like H_2_O_2_) that limit sustained propulsion in physiological environments, potential cytotoxicity from nanomotor accumulation, scalability issues for clinical production, and difficulties in precise navigation through complex biofilm architectures [245]. These motor-based physical strategies provide promising alternatives or complements to chemical and biological antibiofilm approaches, especially for recalcitrant biofilms in medical and industrial settings, by directly targeting the mechanical stability of biofilms, though overcoming translational hurdles remains critical.

Table 2 presents various physical strategies and agents employed for antibiofilm activity, outlining their mechanisms of action and efficacy, used in different bacterial species, highlighting recent advances in the field of biofilm eradication.

**Table 2:** Selected physical antibiofilm agents, their mechanisms of action, and their efficacy.

Strategy	Antibiofilm Agent	Target Bacteria	Mechanism of Action	Efficacy	Reference
**Ultrasound**	High-intensity ultrasound at 20 kHz and 120 W	*S. aureus*	Microbubble collapse and shear forces mechanically disrupt the biofilm matrix	55% reduction in biofilm viability after 1 min	[217]
**Ultrasound + antibiotics**	Low-frequency ultrasound + conventional antibiotics	*S. aureus,* *P. aeruginosa*	Disrupts biofilm matrix; enhances antibiotic penetration	4 h: >99% reduction (*P. aeruginosa*) and 95–97% (*S. aureus*)	[219]
**Ultrasound + AMPs**	Human β-defensin-3 (HBD-3) + (UTMD)	MRSA and MRSE	Enhances the inhibition of biofilm-associated gene expression and reduces biofilm density	Significantly reduced biofilm density and live bacterial counts in vivo	[221]
**Electrical Fields**	Low-amperage DC	*S. aureus,* *S. epidermidis,* *E. coli,* *P. aeruginosa*	Disrupts biofilm and kills cells via electrical stimulation	5.2–5.5 log CFU/cm^2^ reduction	[213]
**Electrical Fields**	Platinum electrodes delivering DC	*S. aureus, S.epidermidis* *P. aeruginosa*	Dose- and time-dependent reduction in viable biofilm cells	Up to complete eradication in 4 days	
**Antimicrobial Photodynamic Therapy (aPDT)**	Photosensitizer: Rose Bengal (RB) + green-light irradiation	*P. aeruginosa* and MRSA	Membranes and biomolecules, leading to cell death	For MRSA: 100% inhibition under irradiation; for *P. aeruginosa*: up to ~37% inhibition (depending on RB concentration and exposure time)	[228]
**Antimicrobial Photodynamic Therapy (aPDT) using porphyrin nano emulsion (NewPS)**	Porphyrin-based nano emulsion (NewPS) + light activation	*S. aureus* biofilms (also tested on planktonic *S. aureus* and *Streptococcus pneumoniae)*	Light-activated porphyrin nano emulsion generates ROS, disrupting bacteria and biofilm matrix.	Up to 6 log_10_ reduction in biofilm bacteria with uniform photosensitizer distribution and extensive cell death	[231]
**Cold Atmospheric Plasma (CAP)**	Gas-phase plasma plume producing reactive species	*S. mutans* dental biofilm	Generation of ROS, oxidative damage of cells and EPS, disruption of biofilm structure, and cell death	Effectively inactivates *S. mutans* biofilm with a log-reduction of 3.08 after 15 min	[235]
**Cold Atmospheric Plasma (CAP) + Antibiotics**	RONS followed by conventional antibiotics	*P. aeruginosa* biofilms	Oxidative damage, increased membrane permeability EPS, enhanced antibiotic penetration	Reduced MICs/MBECs: up to 512-fold reduction for ciprofloxacin/gentamicin and 256-fold	[236]
**Cold Atmospheric Plasma (CAP)**	Cold atmospheric plasma (ionized air, RONS, charged particles, UV)	*S. aureus*, MRSA *S. aureus* biofilms (24, 48, 72 h)	Membrane damage, metabolic disruption, decreased viability; some reduction in biofilm biomass at 72 h	Up to 5.24 log_10_ reduction in viable bacteria after 180 s; metabolic activity reduced by 80–81%, biomass reduction for 72 h biofilms	[237]
**Cold Atmospheric Plasma (CAP)**	(Ar/O_2_ plasma via 8-element LTCC-based linear-discharge array)	*P. fluorescens*	Damage to EPS and cells, membrane disruption, biofilm structural destabilization	>99% reduction in viable bacteria in treated biofilms after ≤60 s exposure; ∼250-fold decrease in CFU in 2-day *P. fluorescens* after 60 s treatment	[238]
**Micro/nanomotor**	Janus Pt-mesoporous silica nanomotor + ficin + vancomycin	*S. aureus*	Self-propulsion penetrates EPS; ficin hydrolyzes matrix; drug release	82% EPS disruption; 96% bacterial killing	[243]
**Micro/nanomotor**	Light/catalysis-driven cascade nanomotors	MRSA, mixed biofilms	Mechanical penetration; ROS generation; enhanced drug delivery	91% biofilm degradation in vitro; >94% bacterial killing in vivo	[244]

While physical methods offer effective means of disrupting biofilm structure, they are often limited by scalability and potential damage to surrounding tissues or materials. As an alternative, biological strategies have emerged, utilizing living organisms or their derivatives, to specifically target and dismantle biofilms in a more targeted and often sustainable manner.

### 5.3. Biological Approaches

Biological antibiofilm strategies apply natural mechanisms to prevent and control biofilm formation, employing agents such as bacteriophages and probiotics.

#### 5.3.1. Bacteriophages

Bacteriophages (phages) are viruses that specifically infect and lyse bacteria, making them a promising biological strategy for disrupting biofilms [246]. Their antibiofilm activity operates through multiple mechanisms, including the degradation of biofilm matrices and direct bacterial lysis [247]. Phages produce enzymes, such as depolymerase, that break down EPS, destabilizing the biofilm structure and facilitating deeper penetration. This disruption allows phages to infect and replicate within bacterial cells, ultimately leading to cell lysis and further biofilm disintegration [13]. Moreover, phage therapy can synergize with antibiotics, a phenomenon known as phage–antibiotic synergy (PAS) [248]. In this context, phages weaken biofilms, enabling antibiotics to penetrate more effectively. Conversely, sublethal antibiotics can enhance phage infectivity by stressing bacterial cells [249]. Multiple research studies support the potential of bacteriophages to control bacterial biofilms. Ref. [250] demonstrated maximum reductions of 4.0–5.5 log colony-forming units on different surfaces. Ref. [251] showed that phage–antibiotic combination strategies consistently enhanced antibiofilm efficacy against multidrug-resistant *P. aeruginosa*, with optimized phage cocktails producing greater log reductions than antibiotics or phages alone, despite the variability in dosing across experimental models. Ref. [252] demonstrated that bacteriophage ɸWL-3 moderately reduces biofilm biomass in *E. coli* (ciprofloxacin/ceftriaxone-resistant) by lysing bacterial cells and disrupting biofilm structure, with a phage concentration of ~10^8^ PFU/mL. When combined with ciprofloxacin or ceftriaxone, bacteriophage ɸWL-3 enhances antibiotic efficacy, leading to a stronger reduction in biofilm formation than either agent alone (Table 3). Phage therapy offers several advantages, particularly its high specificity; phages selectively target pathogenic bacteria without harming beneficial microbiota. They also self-replicate at infection sites, reducing the need for repeated dosing, and demonstrating superior penetration capabilities compared to antibiotics when accessing biofilm barriers [253]. Furthermore, genetically engineered phages can be designed to express biofilm-degrading enzymes or antimicrobial peptides, enhancing their effectiveness [254]. For instance, lytic phages such as SATA-8505 have shown promise against multidrug-resistant *S. aureus* biofilms, while phage-derived enzymes like polysaccharide depolymerase are being explored as potential antibiofilm agents [255]. Despite these advantages, several challenges limit the clinical translation of phage therapy. One significant limitation is the narrow host range of phages; they typically target specific bacterial strains, which necessitates the use of broad-spectrum phage cocktails to effectively address polymicrobial biofilms [256]. Additionally, bacteria can develop resistance through receptor modifications or CRISPR (clustered regularly interspaced short palindromic repeats) systems that enable them to evade phage predation [257]. Delivery challenges also persist due to the complexity of biofilms and physical barriers found on medical device coatings that can obstruct phage access [258]. To overcome these limitations, innovative strategies are being developed, such as nanoparticle-based delivery systems, hydrogel coatings on catheters, and liposome encapsulation to enhance phage penetration and stability [13]. For example, phage-bound AgNPs or magnetic nanoparticles can create channels in biofilms, improving the efficacy of both phages and antibiotics [131]. Future directions for phage therapy include developing broad-spectrum phage cocktails that target diverse biofilm-forming species and stimuli-responsive phages that activate in response to specific biofilm signals such as pH changes or enzymes [259]. Emerging technologies like CRISPR-Cas9 also hold the potential for precisely targeting genes associated with biofilm formation, further expanding the applications of phage therapy [260]. Interestingly, phage therapy has gained increasing recognition in recent years, including FDA approval for compassionate use, underscoring its potential for clinical application [261]. Thus, this therapy represents a powerful and evolving tool in the fight against biofilms, with ongoing advancements aimed at improving host specificity, delivery efficiency, and resistance mitigation to enhance its clinical applicability.

#### 5.3.2. Probiotics

Probiotics are live microorganisms that confer health benefits to the host, particularly by modulating the microbiome and enhancing immune responses [262,263]. As a biological antibiofilm strategy, probiotics prevent and control biofilm formation through competitive exclusion and metabolic interactions [131]. By occupying ecological niches and utilizing available resources, they can inhibit the adhesion and growth of harmful bacteria, thereby reducing biofilm establishment [264]. A key mechanism behind this effect is competitive exclusion, where probiotic strains compete with pathogens for adhesion sites on host tissues or surfaces, effectively limiting biofilm formation [265,266]. For example, *Lactobacillus* spp. and *Bifidobacterium* reduce the adhesion of pathogens like *E. coli* O157:H7 and *S. mutans* by downregulating genes involved in adhesion (e.g., sag, csgB) and biofilm formation, thus preventing biofilm formation in various environments, including the gastrointestinal tract and on medical devices [267]. In addition to competitive exclusion, probiotics can produce antimicrobial substances such as bacteriocins, organic acids, and hydrogen peroxide (H_2_O_2_). These metabolites can inhibit the growth of pathogenic bacteria and disrupt existing biofilms [268]. Bacteriocins can lyse pathogens by creating pores in their cell walls, while H_2_O_2_ can damage pathogenic microcolonies within biofilms [161,269]. Furthermore, probiotics may enhance the host’s immune response by stimulating the production of secretory IgA [270]. For example, probiotics like *Lactobacillus casei* and *L. acidophilus* increase IgA production, enhancing mucosal immunity against biofilm-forming pathogens [270]. Research has highlighted the potential of probiotics to prevent dental plaque formation by inhibiting the growth of cariogenic bacteria. Hence, *Lacticaseibacillus casei* and *L. reuteri* suppress *S. mutans* biofilms by downregulating virulence genes (gtfB, gtfC) [271]. Additionally, in gastrointestinal health, probiotics have proven effective in reducing biofilm-associated infections [272]. Their mechanisms of action are diverse, including the production of inhibitory chemicals [273], reduced bacterial adhesion [274], immune system enhancement [12,275], bacteriocin release, and lactic acid production to disrupt QS [276], suppression of pathogen EPS production, and increased indole production to downregulate QS [167,277]. This approach offers a safe, cost-effective, and environmentally friendly alternative to traditional methods, with reduced risk of resistance development. Nevertheless, the use of probiotics has some limitations. Notably, the effectiveness of probiotics can vary significantly based on strain specificity; not all probiotic strains exhibit antibiofilm properties [278]. Additionally, factors such as environmental conditions, dosage, and timing of administration can influence their efficacy. While probiotics may effectively prevent initial biofilm formation, their ability to disrupt established biofilms is less well understood. As detailed in Table 3, *Lactobacillus rhamnosus* GG biofilm extracts exhibit strong antibiofilm activity against *E. coli*, *S. aureus*, and *P. aeruginosa* by interfering with quorum sensing. A six-fold concentration of these extracts resulted in eradication percentages of 57%, 67%, and 76% for the respective biofilms, along with a nearly complete bactericidal effect (~99.9%) [167]. Similarly, *Bifidobacterium* spp. have demonstrated biofilm inhibition exceeding 30% against *E. coli* and *Salmonella* spp., primarily by producing antimicrobial substances or disrupting QS [279].

The following table summarizes various biological strategies and agents employed for antibiofilm activity, outlining their mechanisms of action, efficacy, and concentrations used in different bacterial species, highlighting recent advances in the field of biofilm disruption.

**Table 3:** Selected biological antibiofilm agents, their mechanisms of action, and their efficacy.

Strategy	Antibiofilm Agent	Target Bacteria	Mechanism of Action	Efficacy	[Concentration]	Reference
**Bacteriophage**	Bacteriophage phT4A (lytic phage)	*E. coli*	Lyses bacteria, disrupts the biofilm, and lowers viable cell numbers.	5.5 log10 reduction (plastic) and 4.0 log10 (stainless steel) after 6 h; formation inhibited ~3.2 log10 at 12 h.	N/A	[250]
**Phage–Antibiotic Combination**	*P. aeruginosa* phages + antibiotics (ciprofloxacin, meropenem, etc.)	MDR/XDR *P. aeruginosa biofilms*	Synergy enhances biofilm disruption and prevents resistance	~3.3–4.7 log10 CFU/cm2 reduction; up to ~5 log10 in optimized conditions	N/A	[250]
**Bacteriophage**	Bacteriophage ɸWL-3	*E. coli* (ciprofloxacin/ceftriaxone-resistant)	Phage lysis of bacterial cells	Moderate biofilm killing	Phage: 108 PFU/mL	[252]
**Bacteriophage + antibiotics**	Bacteriophage ɸWL-3 + ciprofloxacin or ceftriaxone		Phage lysis + antibiotic killing; synergy	Strong biofilm reduction	Phage: 108 PFU/mL; Antibiotics: 0.03–16 µg/mL	
**Probiotics**	*L. rhamnosus* GG biofilm extracts	*E. coli*, *S. aureus* and *P. aeruginosa*	QS disruption	A 6-fold concentration of the extracts achieved 57–76% biofilm eradication and ~99.9% bactericidal effect	*L. rhamnosus* GG biofilm concentration: 107 CFU/mL	[167]
**Probiotics**	*Bifidobacterium* spp.	*E. coli*, *Salmonella* spp.	Produces antimicrobial substances or interferes with quorum sensing.	Biofilm inhibition (>30%)	Not specified	[279]
**Probiotics**	*Lactobacillus* spp.	*S. epidermidis*, *L. monocytogenes*	Competes with pathogenic bacteria for resources	Reduced biofilm formation by >40%	N/A	[37]

## 6. Comparative Evaluation and Challenges in Antibiofilm Strategies

The persistent and resilient nature of biofilms presents a significant challenge across the medical, industrial, and environmental sectors. Bacteria within these communities are protected by a complex network of defense mechanisms, including the EPS matrix, efflux pumps, QS, persister cells, enzymatic degradation of drugs, and genetic exchange, as illustrated in Figure 2 [142]. These mechanisms collectively reduce the penetration and efficacy of conventional treatments, necessitating a multifaceted approach to biofilm eradication.

Table 4 below summarizes the comparative features of antibiofilm interventions, highlighting their mechanisms, advantages, limitations, development stages, and typical applications:

**Table 4:** Comparative table of emerging and conventional antibiofilm strategies, highlighting their mechanisms, advantages, limitations, development stages, and typical applications.

Strategy	Mechanism of Action	Advantages	Limitations	Stage of Development	Application
**Antibiotics**	Inhibit bacterial growth	Easy availability	Resistance development	Clinical	Medical
**Degrading enzymes**	Disrupt the biofilm EPS matrix	Selective, low-toxicity, and enhances other antimicrobials	Stability issues, relatively expensive	Experimental	Food, medical, industrial
**Chelating agents**	Sequester metal ions, destabilizing biofilm structure.	Disrupt biofilm, enhance antibiotic penetration, and reduce resistance	Toxicity at high doses, and needs combination therapy.	Clinical	Medical, industrial
**Antimicrobial peptides**	Disrupt cell membrane, inhibit communication, prevent biofilm, promote dispersion	Broad-spectrum activity and reduce resistance	Poor stability, rapid degradation, and high production costs.	Experimental/Preclinical (nano-delivery)	Medical
**Natural compounds**	Interfere with quorum sensing, inhibit adhesion, and disrupt biofilms	Low toxicity, overcome resistance; consumer acceptance	Inconsistent efficacy, lack of standardization, and allergenic effects.	Various (from traditional use to clinical trials)	Food, oral hygiene, and topical
**Nanotechnology**	Membrane disruption, oxidative stress, and antimicrobial delivery	High effectiveness	Toxicity concerns, high production costs	Preclinical/Emerging clinical	Medical, industrial
**Ultrasound**	Physical disruption of biofilm	Enhances antimicrobial efficacy	Biofilm adaptation, tissue damage, and high equipment cost	Applied research	Medical, industrial
**Electrical field**	Disrupts biofilm integrity, reduces viability and biomass	Non-invasive, scalable potential	Requires biofilm access, complex equipment	Experimental	Medical
**Antimicrobial** **Photodynamic therapy**	Light-activated ROS generation	Non-invasive, repeatable, minimal resistance	Limited penetration; requires sensitizer, photosensitivity	Clinical (specific applications)	Medical
**Cold Atmospheric Plasma**	ROS/RNS and UV disrupt EPS and cells.	Broad-spectrum, fast, residue-free	Limited penetration; device variability.	Preclinical–early clinical	Medical, dental, wound care, surface disinfection
**Micro/Nanomotors**	Autonomous propulsion to penetrate/disrupt biofilms, antibacterial delivery	Deep penetration, active delivery, reduces resistance	Fuel/energy dependence, cytotoxicity, scalability issues	Preclinical (in vitro/animal)	Implants, wounds, catheters
**Bacteriophages**	Infect and lyse bacteria, degrade EPS	Specific, self-replicating, biofilm-penetrating, low side effects	Narrow host range, resistance risk, storage challenges	Experimental/Specialized clinical use	Food, medical
**Probiotics**	Competitive exclusion, antimicrobial production, quorum-sensing disruption	Preventative, consumer accepted, complements other therapies	Strain-specific effects, formulation, and regulatory challenges	Various (dietary supplements to clinical trials)	Medical, industrial

Comparing these strategies reveals distinct efficacy, safety, scalability, and cost patterns. Conventional antibiotics remain the most accessible option, but their effectiveness is increasingly undermined by the rapid evolution of resistance and poor biofilm penetration [125]. Enzyme-based and chelating agents target the structural integrity of biofilms, enhancing the effectiveness of co-administered antibiotics; however, their practical use is often constrained by issues such as low stability, high production costs, and potential toxicity at elevated concentrations [280]. Similarly, antimicrobial peptides and natural compounds provide broad-spectrum, low-toxicity, and resistance-overcoming effects, but stability, standardization, and production costs limit their widespread adoption [23]. Emerging technologies such as nanotechnology, ultrasound, and electrical fields demonstrate high potential for biofilm eradication due to their ability to physically or chemically disrupt biofilm integrity and enhance antimicrobial delivery [281,282]. Despite their potential, these methods are often associated with concerns about toxicity, equipment complexity, and scalability, and most remain in preclinical or experimental stages [280]. Biological strategies, particularly those involving bacteriophages and probiotics, provide highly specific and potentially sustainable solutions. Bacteriophages are capable of self-replication and precise targeting of biofilm-forming bacteria, often penetrating the EPS matrix effectively. However, their narrow host range, the potential for resistance development, and storage difficulties restrict their widespread use. Through mechanisms such as competitive exclusion, antimicrobial production, and quorum-sensing interference, probiotics offer preventive benefits and consumer-friendly profiles. Nonetheless, their application is complicated by strain-specific effects, formulation variability, and regulatory uncertainties [154]. Critically, when directly comparing these classes, chemical agents offer rapid action but generally fail to overcome mature EPS barriers without assistance [5], whereas physical strategies excel at mechanical disruption yet lack specificity and may require costly infrastructure [154]. Biological approaches, while the most precise and ecologically compatible, often suffer from limited host range, environmental sensitivity, and complex regulatory pathways, making them slower to implement at scale [278]. Thus, each category presents a trade-off between efficacy, safety, and practicality, with no single strategy capable of addressing the multifactorial resilience of biofilms on its own. A major challenge across all strategies is the translation of promising laboratory results into clinically effective, safe, and scalable therapies. Many interventions that show significant biofilm reduction in vitro or animal models have yet to demonstrate durable efficacy in human clinical trials, often due to poor biocompatibility, insufficient local concentrations, or the adaptive responses of biofilm communities [51]. Furthermore, the economic burden and regulatory complexity of developing and deploying novel antibiofilm agents slow their integration into routine practice [131]. In summary, while significant progress has been made in diversifying the antibiofilm toolbox, each strategy faces unique challenges related to resistance, delivery, safety, and cost. A multifaceted approach combining chemical, physical, and biological methods, along with continued innovation in drug delivery and regulatory frameworks, is essential to overcome the defenses of biofilm-associated infections and achieve meaningful clinical and industrial impact.

## 7. Future Perspectives

The persistent challenge of biofilm-associated infections demands innovative approaches beyond traditional antimicrobials. Emerging strategies aim to revolutionize biofilm management, shifting from reactive treatment of mature biofilms toward predictive, proactive, and preventive interventions. Recent advancements in 2025, including CRISPR-phage synergies and AI-driven predictive modeling, exemplify this transition [283,284].

CRISPR-based genetic manipulation is a transformative tool, enabling precise silencing of biofilm regulatory genes. CRISPR interference (CRISPRi) targeting *Pseudomonas aeruginosa* suppressed EPS-associated genes, significantly reducing biofilm formation [281]. Future CRISPR strategies aim to integrate reporter genes for real-time biofilm monitoring, engineer beneficial strains that disrupt quorum-sensing networks, and prevent harmful metabolite secretion in food or oral health applications [285].

Engineered bacteriophages complement genetic editing, targeting bacterial cells, degrading EPS, and integrating CRISPR-Cas9 for precise gene disruption [241,286]. Broad-spectrum and stimuli-responsive phages, activated by pH shifts or enzymatic signals, are under development [259]. Phage–nanomaterial conjugates enhance biofilm penetration, while combination approaches with enzymatic or chemical agents synergistically degrade biofilms and prevent regrowth, minimizing host cytotoxicity [283].

AI-driven approaches accelerate biofilm research by predicting pathogen behavior, guiding tailored therapy, and enabling rapid discovery of novel antibiofilm compounds [287,288]. Integration of AI with CRISPR supports sustainable biofilm control in both clinical and food contexts [289].

Smart, responsive materials represent another frontier. Enzyme-responsive nanosystems, pH-sensitive hydrogels, and cell-mimicking phospholipid vesicles enable localized drug release and infection detection [290,291,292]. Switchable surfaces that alternate between antifouling and bactericidal states in response to biofilm signals are being explored for medical and industrial applications [293]. Vaccine strategies using biofilm-specific antigens provide prophylactic potential [283].

Translating these innovations faces challenges. Standardized biofilm testing, representative animal models, and regulatory pathways tailored for antibiofilm agents are lacking. Safety, efficacy, and quality standards must address nanomaterials, responsive surfaces, and combination therapies. The clinical validation of daptomycin for prosthetic joint infections illustrates these translational hurdles [294].

Physical biofilm disruption, including acoustic waves, low-intensity electrical fields, and micro-/nanomotors, complements chemical and biological strategies [294]. Micro- and nanomotors mechanically disrupt biofilms while delivering antimicrobials, circumventing classical resistance mechanisms.

Future antibiofilm strategies will rely on integrated, synergistic systems rather than single interventions. Combining CRISPR, phage therapy, AI-guided selection, smart materials, and physical dispersal is likely the most effective approach. Standardized models and combination therapies, such as phage–nanomaterial conjugates, are urgently needed. Success requires innovative clinical trial designs, regulatory adaptability, and cross-sector collaboration to translate these next-generation therapies from bench to bedside and scalable applications.

**Figure 5:**
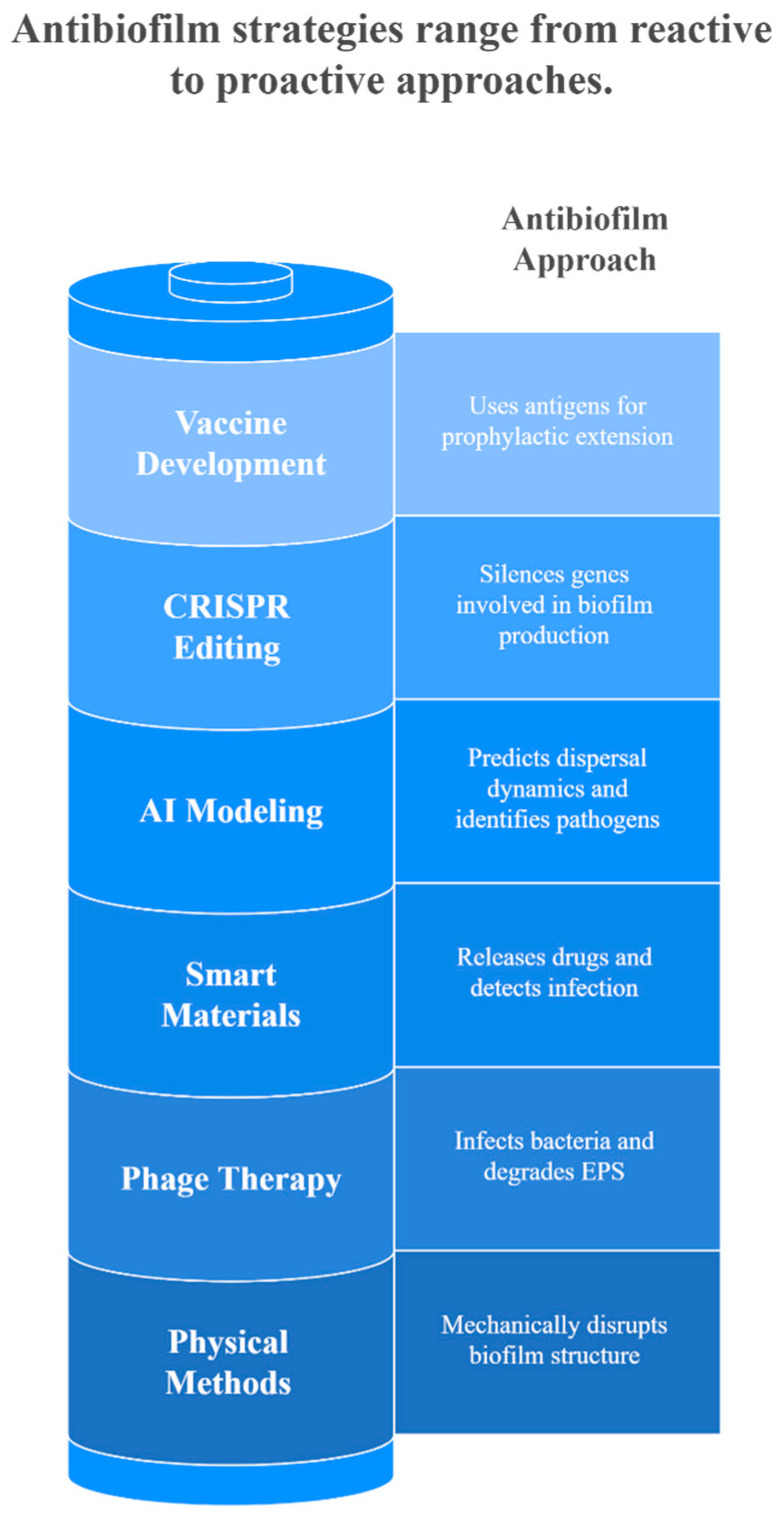
Overview of Emerging Antibiofilm Strategies from Reactive to Proactive Approaches.

Figure 5 illustrates this integrative perspective, synthesizing major antibiofilm strategy classes and their principal modes of action along a continuum from reactive to proactive approaches. The field is moving toward a framework in which prediction, prevention, and multi-modal precision disruption replace traditional reactive treatments of mature, highly tolerant biofilms.

## 8. Conclusions

Biofilms pose a significant challenge in medical, industrial, and environmental fields due to their resilience, adaptability, and ability to confer resistance to antimicrobial agents. This review highlights biofilms’ complex structure and lifecycle, their resistance mechanisms, and the diverse strategies currently employed for their control. A wide array of antibiofilm strategies has been developed, ranging from chemical agents and physical methods to biological approaches such as probiotics, bacteriophages, and innovative nanotechnologies. Despite these advancements, biofilm-associated infections and contamination remain persistent issues, underscoring the need for ongoing research and innovation. Future perspectives in biofilm control should focus on overcoming the limitations of current strategies while applying emerging technologies. Combating biofilms demands an interdisciplinary approach that integrates microbiology, materials science, biotechnology, and clinical research. By optimizing existing strategies and exploring novel technologies, we can move closer to overcoming the challenges posed by biofilms in various settings.

## Data Availability

The original contributions presented in this study are included in the article. Further inquiries can be directed to the corresponding authors.

## Abbreviations

The following abbreviations are used throughout the manuscript for clarity and conciseness.

EPS	Extracellular polymeric substance
CAUTIS	Catheter-associated urinary tract infections
NIH	National Institutes of Health
MDR	Multidrug-resistant
LAB	Lactic acid bacteria
HOCs	Hydrophobic organic compounds
ARGs	Antibiotic resistance genes
eDNA	Extracellular DNA
PNAG	poly-N-acetylglucosamine
QS	Quorum sensing
Pel	Pellicle
Psl	Polysaccharide Synthesis Locus
VBNC	Viable But Non-Culturable
c-di-GMP	Bis-(3′-5′)-cyclic dimeric guanosine monophosphate
PIA	Polysaccharide intercellular adhesin
AI	Autoinducer
AHLs	Acyl-homoserine lactones
AIPs	Autoinducing peptides
AI-2	Autoinducer-2
AI-3	Autoinducer-3
SCVs	Small colony variants
HGT	Horizontal gene transfer
EDTA	Ethylenediaminetetraacetic Acid
DNases	Deoxyribonucleases
AgNPs	Silver nanoparticles
AMPs	Antimicrobial peptides
FDA	Food and Drug Administration
CEO	Clove essential oil
OEO	Oregano essential oil
NPs	Nanoparticles
ROS	Reactive oxygen species
AuNPs	Gold nanoparticles
HIU	High-intensity ultrasound
UTMD	Low-frequency ultrasound-targeted microbubble destruction
MRSA	Methicillin-resistant *Staphylococcus aureus*
MRSE	Methicillin-resistant *Staphylococcus epidermidis*
DC	Direct current
PEF	Pulsed electrical field
aPDT	Antimicrobial Photodynamic therapy
PS	Photosensitizers
RONS	Reactive oxygen and nitrogen species
PAS	Phage–antibiotic synergy
CRISPR	Clustered regularly interspaced short palindromic repeats
H_2_O_2_	Hydrogen peroxide
AI	Artificial intelligence
